# An inhibitory mechanism of AasS, an exogenous fatty acid scavenger: Implications for re-sensitization of FAS II antimicrobials

**DOI:** 10.1371/journal.ppat.1012376

**Published:** 2024-07-15

**Authors:** Haomin Huang, Shenghai Chang, Tao Cui, Man Huang, Jiuxin Qu, Huimin Zhang, Ting Lu, Xing Zhang, Chun Zhou, Youjun Feng

**Affiliations:** 1 Key Laboratory of Multiple Organ Failure, Ministry of Education; Departments of Microbiology and General Intensive Care Unit of the Second Affiliated Hospital, Zhejiang University School of Medicine, Hangzhou, Zhejiang, China; 2 Center of Cryo-Electron Microscopy, Zhejiang University, Hangzhou, Zhejiang, China; 3 School of Life Sciences, Northwestern Polytechnical University, Xi’an, Shaanxi, China; 4 Department of Clinical Laboratory, Shenzhen Third People’s Hospital, National Clinical Research Center for Infectious Diseases, The Second Affiliated Hospital of Southern University of Science and Technology, Shenzhen, Guangdong, China; 5 Cancer Center at Illinois, University of Illinois at Urbana-Champaign, Urbana, Illinois, United States of America; 6 Department of Bioengineering, University of Illinois at Urbana-Champaign, Urbana, Illinois, United States of America; 7 School of Public Health, Zhejiang University School of Medicine, Hangzhou, Zhejiang, China; National Jewish Health, UNITED STATES OF AMERICA

## Abstract

Antimicrobial resistance is an ongoing “one health” challenge of global concern. The acyl-ACP synthetase (termed AasS) of the zoonotic pathogen *Vibrio harveyi* recycles exogenous fatty acid (eFA), bypassing the requirement of type II fatty acid synthesis (FAS II), a druggable pathway. A growing body of bacterial AasS-type isoenzymes compromises the clinical efficacy of FAS II-directed antimicrobials, like cerulenin. Very recently, an acyl adenylate mimic, C10-AMS, was proposed as a lead compound against AasS activity. However, the underlying mechanism remains poorly understood. Here we present two high-resolution cryo-EM structures of AasS liganded with C10-AMS inhibitor (2.33 Å) and C10-AMP intermediate (2.19 Å) in addition to its apo form (2.53 Å). Apart from our measurements for C10-AMS’ Ki value of around 0.6 μM, structural and functional analyses explained how this inhibitor interacts with AasS enzyme. Unlike an open state of AasS, ready for C10-AMP formation, a closed conformation is trapped by the C10-AMS inhibitor. Tight binding of C10-AMS blocks fatty acyl substrate entry, and therefore inhibits AasS action. Additionally, this intermediate analog C10-AMS appears to be a mixed-type AasS inhibitor. In summary, our results provide the proof of principle that inhibiting salvage of eFA by AasS reverses the FAS II bypass. This facilitates the development of next-generation anti-bacterial therapeutics, esp. the dual therapy consisting of C10-AMS scaffold derivatives combined with certain FAS II inhibitors.

## Author summary

An AasS-aided eFA recycling compromises the clinical efficacy of FAS II-directed antimicrobials. The C10-AMS compound is an effective inhibitor for AasS with the Ki value of ~0.6 μM. Efficient binding of AasS by C10-AMS blocks the entry of fatty acyl substrates of various length. The C10-AMS inhibitor mimics an acyl-AMP adenylate to trap AasS in a closed conformation rather than an open state. Collectively, inhibiting a bacterial eFA scavenger AasS enables the re-sensitization of FAS II-targeted antimicrobials. This provides proof of concept that a combination of C10-AMS scaffold derivatives with appropriate FAS II inhibitors constitutes a next-generation anti-virulence biotherapy.

## Introduction

Antimicrobial resistance (AMR) is recognized by the World Health Organization (WHO) as a top 10 challenge to global health and sustainable development, of which the main driver refers to misuse and/or overuse of antimicrobials. WHO declared that AMR-caused global deaths are estimated to rise from ~0.7 million in 2014 [1], to ~1.27 million, in 2019 [2]. Thereby, Jim O’Neil predicted that annual deaths might reach 10 million by 2050 [1,3]. Because of limited efforts to contain AMR spread during the COVID-19 period (from 2019 to 2022), it is expected to worsen partially, which forms a cross-cutting, silent pandemic approaching alarming proportions [4]. As the top priority member of ‘ESKAPE’ pathogens, the resistant *Escherichia coli* (*E*. *coli*) primarily causes life-threatening infections that might pose extensive health/economic impacts over the next decade [5]. The close relative of *E*. *coli*, *Vibrio harveyi* (*V*. *harveyi*) is an opportunistic pathogen of shrimps and invertebrates in marine aquacultures [6]. Multiple drug resistances observed for certain *V*. *harveyi* isolates are due to an ever-increasing number of acquired AMR determinants, like *tetB* and *qnrA* [7–9]. To tackle AMR crisis, a unified ‘one health’ approach is prioritized, comprising multiple sectors of humans, domestic/wild animals, plants and the wild environments (i.e., ecosystems) [10].

**Fig 1:**
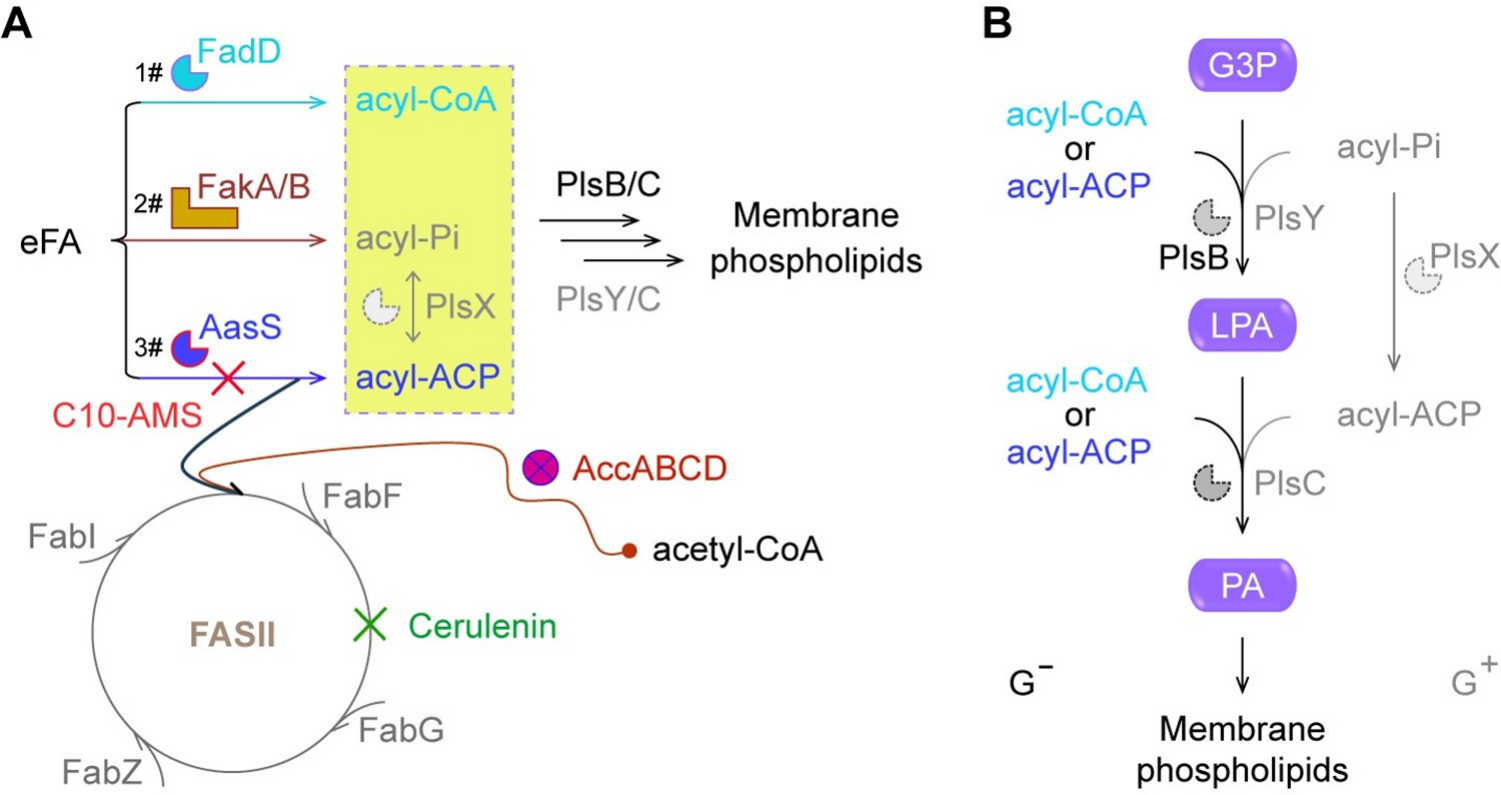
Targeting eFA salvage re-sensitizes bacterial pathogens to killing by cerulenin-included FAS II inhibitors. **A.** eFA salvage coupled with FAS II pathway contributes to membrane phospholipid synthesis. Three mechanisms for eFA scavenging were included here, namely (i) FadD acyl-CoA ligase [29]; (ii) FakA/B system composed of the FakA kinase component and the FakB fatty acid-binding subunit [25,28]; (iii) Acyl-ACP synthetase, AasS [47,48]. Cerulenin denoted the FabF inhibitor targeting a FAS II pathway. **B.** The combination of PlsB/Y-PlsC and PlsX/Y-PlsC represented two alternative routes for the synthesis of membrane phospholipids. The pathway begins with G3P as a recipient, and extends using different primer substrates (acyl-CoA/acyl-ACP for PlsB/PlsC vs acyl-Pi for PlsY/X, and acyl-ACP for PlsC). Abbreviations: C10-AMS, 5’-O-(N-decanylsulfamoyl) adenosine; FadD, Acyl-CoA ligase; FakA/B, Fatty acid kinase A in complex with fatty acid-biding subunit B; AasS, acyl-ACP synthetase; AccABCD, Acetyl-CoA carboxylase composed of four subunits (namely (i) AccA, α-subunit of carboxyltransferase; (ii) AccB, biotin carboxyl carrier protein (BCCP); (iii) AccC, biotin carboxylase (BC); and (iv) AccD, β-subunit of carboxyltransferase); FAS II, Type II fatty acid synthesis pathway; FabI, Enoyl-ACP reductase; FabF, β-ketoacyl-ACP synthase II; FabG, Ketoacyl-ACP reductase; FabZ, 3-hydroxyacyl-ACP dehydratase; G3P, Glycerol-3-phosphate; LPA, Lyso-phosphatidic acid; PA, Phosphatidic acid; acyl-Pi, acyl phosphate; PlsB, G3P acyltransferase; PlsY, Acyl-Pi-dependent G3P acyltransferase; PlsX, Phosphate: acyl-ACP transacylase; PlsC, LPA acyltransferase; G^-^, Gram-negative bacterium; G^+^, Gram-positive bacterium.

Fatty acids (FA) are a group of energetically-expensive building blocks for membrane phospholipid synthesis in the tree of life. Unlike eukaryotic cells with Type I Fatty Acid Synthesis (FAS I) machinery, a giant multienzyme complex, diverse bacterial species exploit the type Il FAS system (FAS II), consisting of multiple discrete subunits [11]. The essentiality of the two FAS II metabolites [i.e., β-hydroxyl FA in Gram-negative bacterium [12], and pentadecanoic acid, a branched-chain FA in Gram-positive pathogen [13]] enables the possibility of the FAS II machinery as a druggable pathway. As expected, FAS II-directed mining of natural products led to a repertoire of attractive lead compounds. Namely, they include, but not limited to (i) cerulenin [14–16] and platensimycin [17], two selective FabB/F inhibitor (**Fig 1A**); (ii) platencin, a natural product with dual targets FabH and FabB/F [18]; and (iii) an arsenal of FabI-targeted antimicrobials (i.e., triclosan, a widely-used biocide [19–21]; isoniazid, the front-line anti-TB drug [22]; and *Staphylococcus*-specific AFN1252 [23]). Apart from the FAS II pathway, the majority of bacterial pathogens develop diverse mechanisms to recycle environment/exogenous FA (eFA) [12,24]. Unlike Gram-positive pathogens (*Staphylococcus* [25,26], and *Streptococcus* [27,28]) having fatty acid kinase FakAB systems, Gram-negative bacterium relies on either acyl-CoA ligase FadD [29] or acyl-ACP synthetase (Aas) exemplified with the *E*. *coli* bifunctional Aas [30–32] and its relic AasC of *Chlamydia* [33]. This raises the possibility that an eFA salvage compromises the effectivity of FAS II inhibitors by replacing *de novo* synthesized FAs (**Fig 1**) [34–36]. In fact, *S*. *aureus* liberates host FAs from abundant low-density lipoproteins (LDL) [37], and the assimilated eFA favors staphylococcal anti-FAS II adaptation at the infection site [13,38]. In addition, staphylococcal FAS II bypass is in part, if not all, traced to the polymorphism of *fabD* [39] and *acc* [40], two essential genes that initiate a FAS II pathway [41]. A growing body of evidence explains the failure of FAS II antibiotic-based anti-MRSA therapy in a mouse bacteremia model [38–40]. Combined with (p)ppGpp inducer of stringent response, FAS II antimicrobials can block MRSA outgrowth, offering a synergistic bi-therapy strategy [42]. Because staphylococcal anti-FAS II bypass is engendered via an eFA scavenging by the FakAB system [37,38], it is in rational to formulate an alternative bi-therapeutics by using the anti-FAS II drug AFN1252 mixed with some FakAB inhibitor. Finally, we are eager to find out whether or not an Aas enzyme mimicking FakAB machinery exclusively in G-positive pathogens can confer G-negative bacterial adaptation to FAS II-directed antimicrobials.

The Aas members belong to a ubiquitous group of acyl-activating enzyme (AAE), also called adenylate-forming enzyme. The *in vitro* Aas activity is originally traced to the *E*. *coli* bifunctional 2-acyl-glycerolphosphoethnomine (2-acyl-GPE) acyltransferase/acyl-ACP synthetase [30]. The physiological role of the *E*. *coli* Aas enzyme denotes the ligation of an activated fatty acyl chain from acyl-ACP intermediate to the 1-position of Lyso-phospholipid (LysoPL), a byproduct of lipoprotein synthesis [32,43]. Because the resultant acyl-ACP intermediate is tightly bound by the Aas enzyme, it cannot access the membrane phospholipid/lipopolysaccharide (LPS)-lipid A pathway [12,24]. Indeed, the interfacial Aas of *E*. *coli* displays an *in vitro* ‘artifact’ salt-dependent activity of synthesizing acyl-ACP thioester [30,32]. In contrast, the marine bioluminescent bacterium *V*. *harveyi* AasS (VhAasS, thereafter called AasS) is a cytoplasmic version of acyl-ACP synthetase [44–46]. Notably, this soluble AasS channels a pool of eFA nutrients to build bacterial phospholipids as well as LPS-lipid A (**Fig 1A** and **1B**) [47,48]. This paradigmatic VhAasS is featured by its substrate promiscuity and is leveraged as a versatile tool in synthetic biology [49–51]. So far, the toolbox of Aas enzymes contains four additional members capable of eFA assimilation. Namely, they include (i) SynAas of Cyanobacteria [52,53] that is partially equivalent to AAE15, a cousin of the plant *Arabidopsis* [54]; (ii) AasC of the sexually-transmitted, obligate intracellular parasite *Chlamydia trachomatis* [33]; (iii) AasN exclusively in the human pathogens *Neisseria meningitis* and *N*. *gonorrhoeae* [55]; and (iv) two isoforms (AfAas1 for C12:0 & AfAas2 for C18:1) arising from *Alistipes finegoldii*, a representative resident in human gut microbiomes [56]. Very recently, an extensive cryo-EM study revealed that unlike all the other AAE members with known structures (e.g., ttLC-FACS dimer [57]), AasS acts as ring-shape hexamer, and adopts a ‘conformational rearrangement’ pattern to execute its catalysis cycle [58]. The gating role of W230 is functionally defined in the context of AasS action, allowing bacterial salvage of eFA nutrients [58]. This represents the first structural landscape for acyl-ACP synthetase of versatility. It is an open question to ask if the other pathogen cousins of AasS (such as AasC [33] and AasN [55]) also feature a similar structural architecture.

The annual *V*. *harveyi* infection leads to a substantial economic loss in aquacultural production. Combined with certain FAS II antimicrobials, development of AasS inhibitors, opens perspectives of a dual-therapy against marine pathogens. Recently, the lead compound, 5’-O-(N-decanylsulfamoyl) adenosine (termed as C10-AMS) was found to efficiently impair the activities of AasS [59] and its paralogs like AasN/AasC (**Fig 1A**) [60]. Despite that it assumed to mimic the decanoyl-adenylate intermediate (abbreviated as C10-AMP), how the broad inhibitor C10-AMS antagonizes AasS-based eFA recycling is largely unclear. We solved two high-resolution cryo-EM structures of AasS liganded with C10-AMS inhibitor (2.33 Å) and C10-AMP intermediate (2.19 Å) in addition to its apo form (2.53 Å). Structural and biochemical comparison explains how the inhibition of AasS proceeds by the mixed-type inhibitor C10-AMS. Altogether, this study constitutes a proof of concept for inhibiting an eFA scavenger AasS (and/or its isoform AasN/AasC) to overcome bacterial bypass of anti-FAS II antimicrobials.

## Results and discussion

### The AasS bypass of a druggable FAS II pathway

The FAS II pathway is a conserved mechanism for bacterial *de novo* fatty acid synthesis, and provides diverse acyl chains for building phosphatidic acid, a precursor of membrane phospholipid synthesis (**Fig 1**). The acetyl-CoA carboxylase (AccABCD) initiates the first-committed step of a FAS II pathway [61], giving malonyl-CoA, a cognate substrate for FabD (malonyl-CoA: ACP transacylase) [62–64]. The resultant malonyl-ACP acts as a primer with the destination to enter a FAS II cycle consisting of four iterative steps (**Fig 1A**). Namely, these include (i) condensation of malonyl-ACP by FabH (β-ketoacyl-ACP synthase III) to 3-ketobutyryl-ACP intermediate [65–67]; (ii) reduction of β-ketoacyl-ACP by FabG (β-ketoacyl-ACP reductase) [68,69]; (iii) dehydration of β-hydroxyacyl-ACP by FabZ (β-hydroxyacyl-ACP dehydratase) [70–72]; and (iv) reduction of enoyl-ACP by FabI (enoyl-ACP reductase) [73–75]. Unlike FabH that is restricted to an initial condensation [65–67], the FabF (β-ketoacyl-ACP synthase II) specifically directs those β-ketoacyl-ACP species elongated with a 2-carbon unit per cycle to reenter a FAS II route (**Fig 1A**) [76,77].

In contrast to the two known systems (acyl-CoA synthetase FadD [12,29], and FakA/B kinase complex [25,28,78]), the AasS acyl-ACP synthetase represents a third mechanism for eFA recycling, which circumvents a druggable FAS II machinery (**Fig 1A**). This is because long-chain (LC) acyl-ACP thioesters loaded by AasS from exogenous fatty acids replace partially the nascent acyl species to participate in the formation of phosphatidic acids. In *E*. *coli*, LC acyl-CoA (rather than acyl-ACP) is acylated on the 1 and 2-positions of glycerol-3-phosphate (G3P) by the PlsB/PlsC acyltransferase system (**Fig 1B**) [12,24]. Nevertheless, the LC acyl-ACP ester that arises from activation of eFA pools by certain AasS isoenzyme (AasC [24,33] and AasN [55]), is assumed to initiate phospholipid synthesis in certain human pathogens. Not surprisingly, the kind of activated acyl form is also converted by PlsX (phosphate: acyl-ACP acyltransferase) to acyl-phosphate (acyl-P) intermediate [55,79], a canonical substrate for PlsY, an acyl-P dependent G3P acyltransferase (**Fig 1A**) [79,80]. This is because the PlsX/Y pair is dominant in relative to the PlsB/C system. Unlike the Gram-positive bacterium that harbors Fak-PlsX/Y machinery and exploits acyl-P as a switcher for phosphatidic acid synthesis [25,79], the Gram-negative organism carrying Aas-PlsX/Y system, generally adopts acyl-ACP esters as an initiator for membrane phospholipid formation (**Fig 1B**) [11,55]. Given that the AasS route for eFA acquisition circumvents the FAS II-directed inhibitor, esp., cerulenin, harnessing an AasS-targeted antibacterial agent (e.g., C10-AMS) is plausible to reverse such a FAS II bypass (**Fig 1A**).

### Inhibition of AasS by C10-AMS

**Fig 2:**
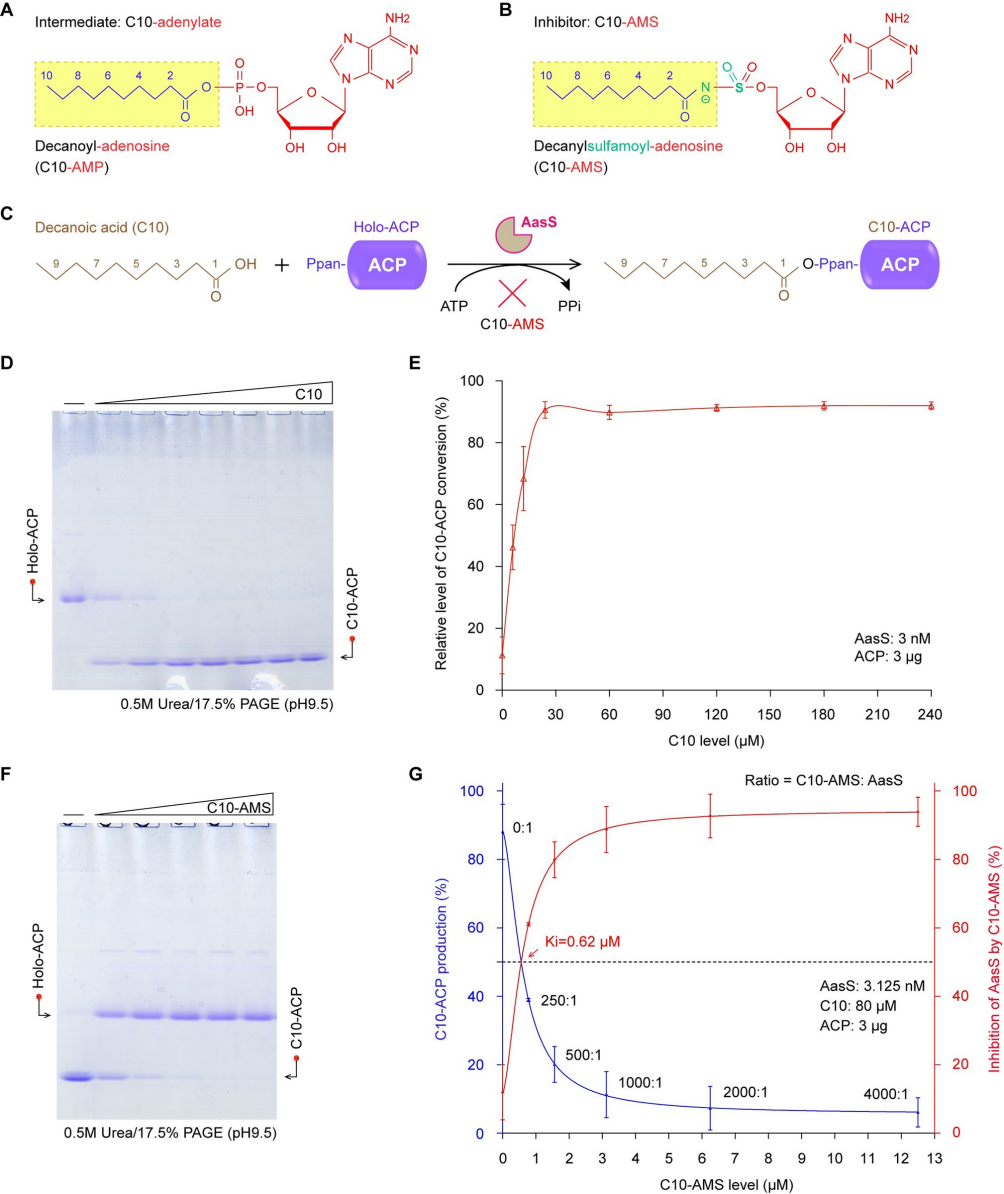
AasS activity with C10 fatty acid substrate is abolished by the C10-AMS inhibitor. Chemical structures of an intermediate C10-adenylate (**A**) and the inhibitor C10-AMS (**B**). **C.** The principle for ligation of decanoic acid (C10) by AasS with holo-ACP to generate C10-ACP, and its inhibitory mechanism by C10-AMS. Qualitative analysis (**D**) and relative quantitation curve (**E**) of C10-ACP conversion from C10 substrate in a dose-dependent manner. In the reaction system of AasS (3 nM), unlike the ACP acceptor protein that was added at the constant level of 3 μg (panels D&E), the level of C10 fatty acid varied markedly (6, 12, 24,…, to 240 μM). **F.** The AasS-catalyzed production of C10-ACP *in vitro*, is abolished by C10-AMS inhibitor in a dose-dependent pattern. The conformationally-sensitive gel of 0.5 M urea/17.5% PAGE (pH9.5) was utilized to separate C10-ACP from its acceptor holo-ACP (panels D&F). **G.** Semi-quantitative assays for inhibition of AasS catalysis by C10-AMS compound. The determined Ki of C10-AMS vs AasS is 0.62 μΜ. Except for the C10 substrate that was fixed at 80 μM, AasS reaction was established identically as described in Fig 2D and 2E. It was noted that the ratio of C10-AMS inhibitor to AasS varied dramatically (ranging from 0:1, 250:1, 500:1, 1000:1, 2000:1, to 4000:1). Designations: ACP, acyl carrier protein; ATP, adenosine triphosphate; PPi, pyrophosphate.

The decanoic acid (C10) as a favorable substrate, can be converted into C10-AMP by AasS enzyme [48,58] (**Fig 2A**). Since C10-AMS compound resembles this C10-AMP intermediate (**Fig 2B**), Currie and colleagues preliminarily presented its inhibition of AasS activity *in vitro* [59] (**Fig 2C**). Whereas it awaited further experimental evidence to finely characterize its inhibitory mechanism. As expected from our separation approaches with conformationally-sensitive urea gel (**Fig 2D** and **2E**), almost full activity with C10 fatty acid substrate was assigned to AasS enzyme at the concentration of only 3.125 nM, much lower than 0.5 to 2.0 μM used by Jaremko’s group [59,60]. Also, the C10 acyl substrate added as low as 12 to 24 μM (rather than 1 mM) is sufficient to saturate AasS enzyme (**Fig 2E**). The C10-AMS inhibitor was shown to work well in a dose-dependent manner (**Fig 2F**), and its inhibition constant (Ki) value was measured to be around 0.62 μM (**Fig 2G**). We thus speculated that C10-AMS competes with C10 substrate (or C10-AMP intermediate) for binding to functional cavity of AasS (**S1A Fig**).

To ascertain this hypothesis, we set up three combinations of AasS reaction with the varied order of C10-AMS inhibitor added in relative to C10 substrate. Namely, these included (i) addition of C10-AMS after C10 pre-incubation (**S1B Fig**), (ii) simultaneous addition of C10-AMS and C10 (**S1C Fig**), and (iii) supplementation of C10-AMS prior to C10 (**S1D Fig**). Obviously, a pre-incubation of C10-AMS exhibited appreciable stronger level of inhibiting AasS enzyme (**S1D Fig**), when compared to the other two treatments (**S1B** and **S1C Fig**). The semi-quantitative analyses displayed three distinguishable Ki values, namely (i) 0.67 μM for condition 1#, (ii) 1.37 μM for condition 2#, and (iii) 2.13 μM for condition 3# (**S1E Fig**). Next, we generated Michaelis-Menten curves of AasS enzyme to evaluate the inhibitory pattern of C10-AMS against ATP cofactor and C10 acyl substrate (**S2 Fig**). With the presence of increasing amount of C10-AMS while varying the ATP concentration, the Michaelis constant (Km) of AasS was observed to increase, and its maximum velocity (Vmax) declined (**S2A** and **S2B Fig**). This suggested that C10-AMS inhibitor could adopt a mode of mixed inhibition against ATP ligand. Similarly, the inhibitory pattern of C10-AMS was also likely to be a mixed type of inhibition for the C10 acyl substrate (**S2C** and **S2D Fig**). Retrospectively, a similar scenario has been reported for its analog, OSB-AMS that functions as a mixed-type inhibitor of MenE, an o-succinylbenzoyl-CoA synthetase [81,82]. In the context of inactivation for an AasS-catalyzed eFA recycling pathway (**Fig 1A**), C10-AMS is assumed to behave in a manner of mixed inhibition, rather than the competitive inhibition initially proposed by Currie *et al*. [59].

### Binding of AasS by C10-AMS inhibitor

**Fig 3:**
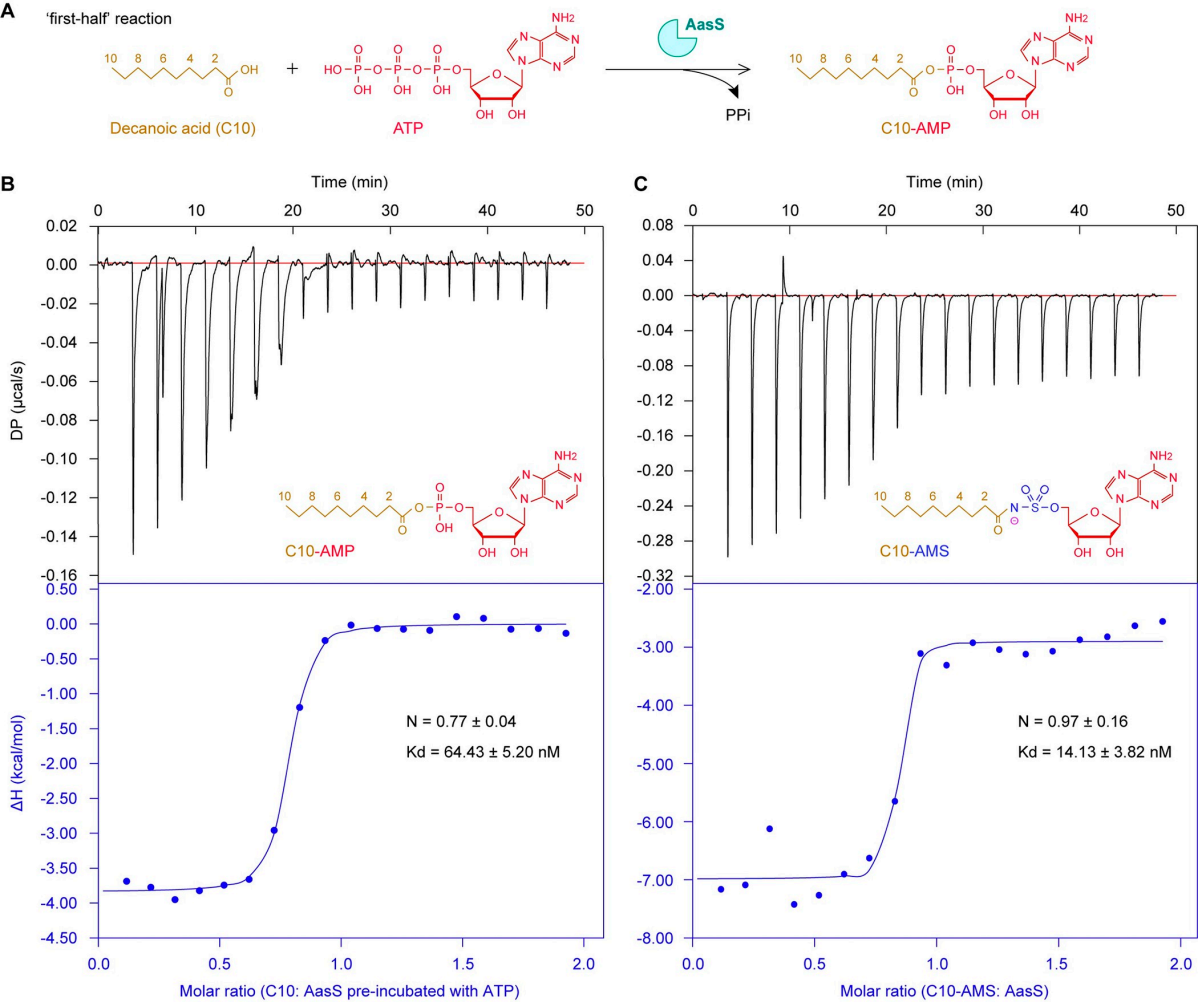
ITC analysis for binding of C10-AMS inhibitor to AasS enzyme. **A.** Scheme for an intermediate of C10-AMP generated from the C10 fatty acid substrate via the ‘first-half’ reaction of AasS activation. **B.** Use of ITC to measure an interaction between AasS enzyme and its C10-AMP intermediate. **C.** ITC-based assay for AasS specifically bound by the C10-AMS inhibitor. Designations: ITC, isothermal titration calorimetry; N, stoichiometry; Kd, dissociation constant; DP, differential power; ΔH, enthalpy.

To examine the binding affinity of C10-AMS inhibitor, we carried out an extensive isothermal calorimetry (ITC)-based investigation. Given that C10-AMP adenylate is produced in the ‘first-half’ reaction of AasS catalysis, ATP molecules were preincubated with the AasS sample before the C10 titration proceeded (**Fig 3A**). In contrast to malic acid and pimelic acid, the two dicarboxylic acids that cannot be recognized by AasS enzyme [48,83], mono-ethyl pimelic acid (E-C7) functions as a surrogate substrate [58,83]. As expected from our ITC assays, unlike a scenario observed for E-C7 [58], none of the two dicarboxylic acids, i.e., pimelate (**S3A Fig**) and malic acid (**S3B Fig**), was titrated with the AasS partner. It was fully consistent with their inability of both being ligated with the ACP vehicle (**S4A Fig**), and replacing E-C7 in the biotin bypass *in vivo* (**S4B** and **S4C Fig**). Also, this validated the efficacy of our ITC performance to some extent. Distinct from the recent notion that an E-C7 substrate binds to AasS with a mild affinity [58], the C10-AMP intermediate exhibits a robust activity for AasS binding in our ITC experiments (**Fig 3**). Apart from its stoichiometry (n) that equals to ~0.77, close to the theoretical value of 1.0, the dissociation constant (Kd) value of C10-AMP was measured to be 64.43±5.20 nM (**Fig 3B**). This is plausible because the C10 fatty acid (rather than E-C7) is an optimal substrate for AasS [48]. A similar scenario was also seen with the C10-AMS inhibitor in that it features the ‘n’ value of 0.97±0.16 (**Fig 3C**), suggesting the molar ratio of C10-AMS vs AasS is 1:1. Whereas, in comparison with the C10-AMP intermediate, this C10-AMS inhibitor seemed to bind more tightly AasS protein, because its Kd value is 14.13±3.82 nM, significantly smaller than the equivalent of C10-AMP (64.43±5.20 nM, **Fig 3C**). Overall, we believed that efficient binding of C10-AMS to AasS is a prerequisite for blocking AasS-dependent eFA salvage.

### Blocking of biotin bypass by C10-AMS compound

**Fig 4:**
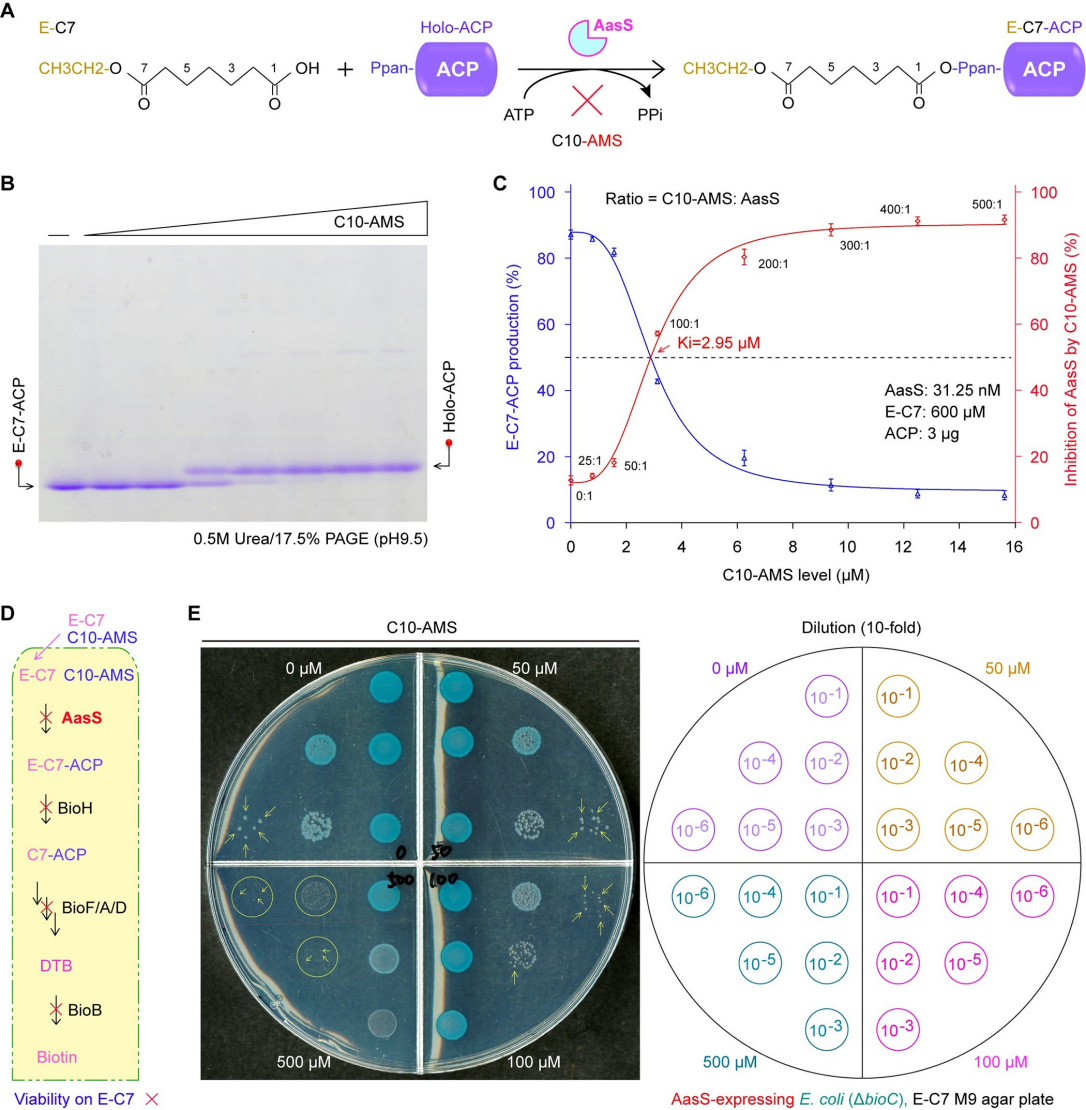
Integrated evidence for the inhibition of AasS action with E-C7 fatty acid by C10-AMS compound. **A.** Schematic diagram of AasS-catalyzed ligation of E-C7 with holo-ACP recipient to produce E-C7-ACP. **B.** Use of conformationally-sensitive PAGE to visualize the fact that the C10-AMS inhibitor displays dose-dependent inhibition on the conversion of E-C7-ACP ester from its acceptor holo-ACP. A representative photograph of three independent trials was given. **C.** Relatively-quantitative analysis (for the inhibitory efficacy of C10-AMS on AasS-catalyzed E-C7-ACP production. As for AasS reaction (panel **B**), unlike the three components that are at constant levels, namely (i) AasS (31.25 nM), (ii) E-C7 (600 μM), and (iii) ACP (3 μg), C10-AMS inhibitor was supplemented at varied ratio in relative to the AasS enzyme (ranging from 0:1, 25:1, 50:1, 100:1, 200:1, 300:1, 400:1, to 500:1). The ImageJ software was applied to measure the relative percentage (%) of E-C7 fatty acylation in each AasS reaction. The resultant graphs were plotted from three independent experiments, in which the output was expressed as means ± SD (standard deviations). **D.** Schematic diagram for C10-AMS inhibition of AasS-based bypass of biotin requirement by the *E*. *coli* Δ*bioC* biotin auxotroph on the non-permissive condition. **E.** Altered viability of AasS-expressing *E*. *coli* Δ*bioC* strain suggested the *in vivo* inhibition of C10-AMS in a dose-dependent manner. Using M9 defined medium with E-C7 as sole carbon source (displayed in panel on left hand), biotin bypass assays were performed with the Δ*bioC* strain that produces AasS enzyme carried by a plasmid. Log-phase cultures in a series of 10-fold dilution (shown in panel on right hand), were spotted on the x-gal-containing M9 agar plates supplemented with C10-AMS inhibitor at varied levels (0, 50, 100, to 500 μM). The addition of x-gal substrate enabled the occurrence of blue colonies for better photographing, and viable colonies were highlighted with yellow arrows.

As earlier described by Lin and Cronan [83,84], the Δ*bioC* mutant of *E*. *coli* requires biotin for its viability. The ability that AasS enzyme channels exogenous E-C7 to give E-C7-ACP (**Fig 4A**), enables circumventing a ‘BioC-BioH’ primary step of biotin synthesis in the Δ*bioC* mutant [58,83]. It is of possibility that C10-AMS blocks the bypass of biotin biosynthesis by AasS. To examine this assumption, we sought experimental evidence *in vitro* and *in vivo*. As shown in our assay with conformationally-sensitive urea gels, the conversion of E-C7-ACP by AasS is inversely proportional to C10-AMS addition (**Fig 4B**). This largely agreed with the reduction of AasS activity with C10 by C10-AMS inhibitor (**Fig 2F**). The Ki value of C10-AMS was determined to be about 2.95 μM for E-C7 acyl substrate (**Fig 4C**). The *in vitro* inhibition of E-C7 utilization by C10-AMS compound promoted our efforts to ascertain its potential of blocking biotin bypass *in vivo* (**Fig 4D**). Indeed, an introduction of plasmid-borne AasS into the biotin auxotroph of *E*. *coli* Δ*bioC* mutant, engendered its occurrence on non-permissive condition of M9 defined agar plates with E-C7 as sole carbon source (**Figs 4E** and **S4B**). However, a replacement for E-C7 carbon source with either malic acid (**S4B Fig**) or pimelic acid (**S4C Fig**), led to loss of function in supporting bacterial viability of the *E*. *coli* Δ*bioC* mutant on the biotin-deficient M9 medium, albeit expression of AasS. As expected from bacterial viability, supplementation of C10-AMS inhibitor at the level of over 100 μM, markedly impaired AasS-based bypass of biotin synthesis in the context of biotin-requiring Δ*bioC* mutant (**Fig 4E**). The accumulated evidence demonstrated that C10-AMS inhibitor can physiologically inactivate AasS bypass of biotin synthesis [83].

### Complex structure of AasS liganded with C10-AMS inhibitor

**Fig 5:**
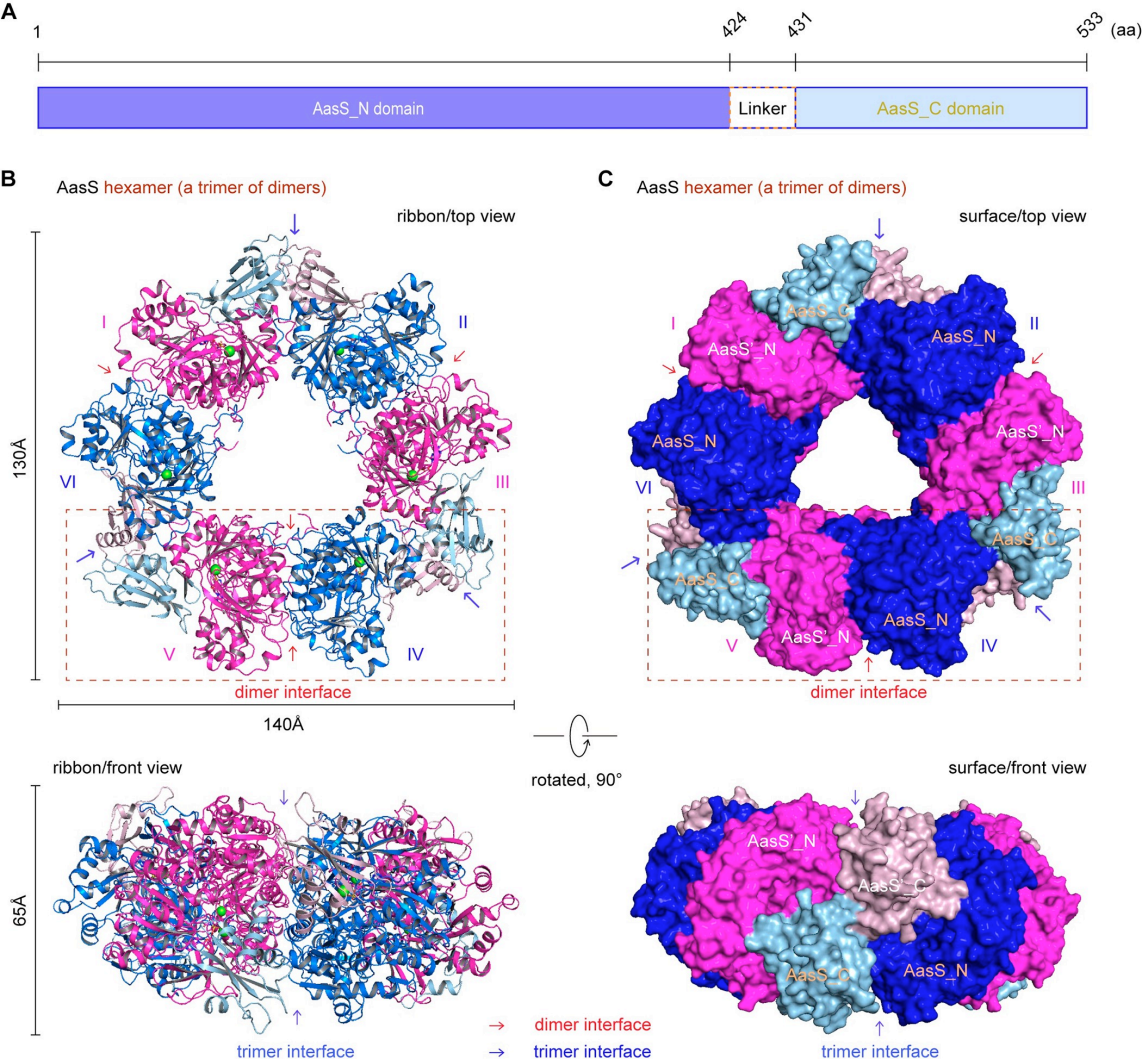
Structural characterization of AasS complexed with its inhibitor C10-AMS. **A.** Linear presentation of full-length AasS enzyme composed of two domains. The large domain AasS_N (residues 1–424) is connected by a short linker (residues 425–430) with the compact small domain, AasS_C (residues 431–533). Ribbon illustration (**B**) and surface structure (**C**) of AasS hexamer liganded with C10-AMS inhibitor. The AasS hexamer (130 x 140 x 65 Å) essentially behaves as a trimer of dimers, in which the subunit of monomer is numbered from I, II, …, to VI. The dimer interface was highlighted with a red arrow, and the trimer interface was indicated with a blue arrow. The AasS top view (130 x 140 Å, in upper panel) was rotated 90° counter-clockwise, giving its front view (65 x 140 Å, in bottom panel). The N-terminal domain of AasS was colored blue for AasS_N (or magenta for AasS’_N), and the C-terminal domain of AasS was displayed in powder blue for AasS_C (or light pink for AasS’_C).

**Fig 6:**
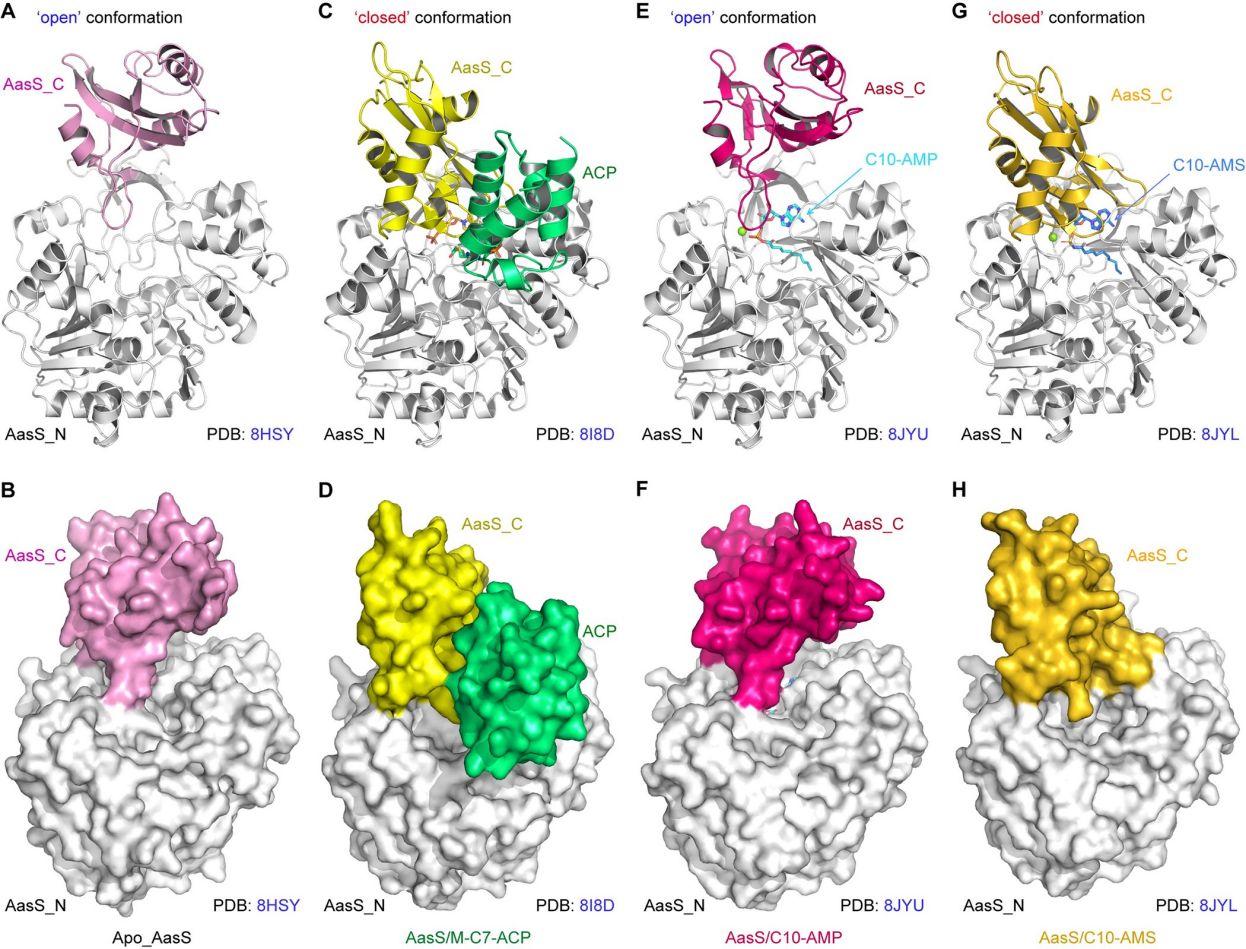
The binding of C10-AMS inhibitor promotes a markedly-conformational rearrangement of AasS enzyme. **A-B.** Ribbon and surface representation of AasS protomer in an apo-form (PDB: 8HSY). As for apo AasS, its AasS_C domain colored pink, appears in an ‘open’ orientation. **C-D.** Cartoon and surface structure of AasS protomer in complex with its product M-C7-ACP (PDB: 8I8D). In this complex, the AasS_C part colored yellow, displays a ‘closed’ orientation. The ACP partner is colored lime-green. Ribbon structure (**E**) and surface illustration (**F**) of an AasS enzyme liganded with a molecule of C10-AMP intermediate per protomer (PDB: 8JYU). The occupancy of C10-AMP intermediate renders the AasS_C domain (colored hot-pink) in an ‘open’ orientation. The C10-AMP molecule is given in a stick representation, whose carbon atoms are colored cyan. **G-H.** Cartoon and surface characterization of C10-AMS inhibitor-liganded AasS protomer (PDB: 8JYL). The effective binding of C10-AMS inhibitor leads to a ‘closed’ orientation of AasS_C domain (in gold) converted from its “open” orientation. Like a C10-AMP molecule, the C10-AMS inhibitor is also given in a stick representation, of which carbon atoms are colored marine. Designations: AasS_C, C-terminal domain of AasS; AasS_N, N-terminal domain of AasS.

Very recently, the AasS (533 aa) of *V*. *harveyi* was found to feature a large domain at N-terminus (termed AasS_N) that is connected with a compact C-terminal domain by a flexible linker (**Fig 5A**) [58]. To gain structural insights into the inhibitory mechanism of AasS action, we solved three sets of single particle cryo-EM structures of AasS at high-resolution (**Table 1**). In addition to the two controls we introduced, (i) AasS alone (**S5** and **S6 Figs**) and (ii) its complex with C10-AMP adenylate (**S7** and **S8 Figs**), we focused on the complex of AasS liganded with C10-AMS inhibitor (**S9 Fig**). As expected, a hexamer ring-like overall structure was invariantly formed in all the three types of AasS particles, regardless of a cofactor/inhibitor. Unlike the two controls that are characterized with a dimension of ‘140x150x80 Å’ (**Figs 5**, **S6** and **S8**) [58], the inhibitor-bound AasS architecture is relatively compact, because that its dimension is only confined to ‘130x140x65 Å’ (**Fig 5B** and **5C**). This raised the possibility that a configurational alteration of AasS is induced by C10-AMS inhibitor. As for each protomer, overall structure of AasS_N (and/or AasS_C) is similar to the counterpart of other known AAE elements (**Fig 6**) [85,86]. In brief, the pitcher-like AasS_N domain consists of three β-sheets flanked by α-helices, and provides a centrally-localized cavity for substrate entry. Whereas, the small cap-like AasS_C domain contains one two-stranded and one three-stranded β-sheets surrounded by three α-helices, hovering on top of the AasS_N active sites (**Fig 6**). Obviously, the pattern of AasS oligomerization is recognized as a trimer of dimers, which mainly relies on two interfaces constituted by the AasS_N domain (**Figs 5**, **S6** and **S8**).

**Table 1:** Cryo-EM Collection, refinement and validation statistics of AasS enzyme liganded with C10-AMS inhibitor or C10-AMP intermediate.

Ligands	AasS		
	Apo-form	C10-AMS	C10-AMP
Data collection and processing			
Magnification	29,000	130,000	130,000
Voltage (kV)	300	300	300
Electron exposure (e^-^/Å^2^)	78	52	52
Defocus range (μm)	1.5~2.5	0.8~1.6	0.8~1.6
Pixel size (Å)	1.014	0.93	0.93
Symmetry imposed	D3	D3	D3
Micrographs	2,329	3,058	3,339
Initial particle images (no.)	1,040,351	1,160,261	1,737,985
Final particle images (no.)	539,382	212,195	602,383
Map resolution (Å) (global)	2.53	2.33	2.19
FSC threshold	0.143	0.143	0.143
Map-sharpening *B* factor (Å^2^)	-84	-89.4	-89.6
Refinement			
PDB code	8HSY	8JYL	8JYU
Model resolution	2.6	2.5	2.3
FSC threshold	0.5	0.5	0.5
Model resolution range (Å)	2.5~38	2.3~45	2.2~36
Model composition			
Non-hydrogen atoms	25320	25434	25542
Protein residues	3180	3162	3180
Ligands	0	12	18
*B* factors (Å^2^)			
Protein	51.68	40.89	73.50
Ligand	N/A	20.61	44.98
R.m.s. deviations (PHENIX)			
Bond lengths (Å)	0.005	0.005	0.005
Bond angles (°)	1.022	1.000	0.987
Validation			
Mol Probity score	1.84	1.47	1.67
Clash score	7.52	8.64	6.37
Poor rotamers (%)	0.80	0.61	0.14
Ramachandran plot			
Favored (%)	93.56	98.13	95.49
Allowed (%)	6.25	1.87	4.32
Disallowed (%)	0.19	0.00	0.19

It is long settled that the paradigm AAE member, 4-chlorobenzoate: CoA ligase (CBL) catalysis involves the transition of two conformations, namely (i) adenylate-forming (open) conformation, and (ii) thioester-producing (closed) conformation [87]. A similar scenario was recorded in the structural rearrangement of AasS, an additional AAE member [58]. In fact, the apo-form of AasS (PDB: 8HSY) here, was captured to adopt an open conformation (**Fig 6A** and **6B**). This is largely opposite to the complex of AasS with its product M-C7-ACP (PDB: 8I8D), because that it is situated in a closed conformation (**Fig 6C** and **6D**). Not surprisingly, like the apo-AasS in a state ready for the adenylation (**S10A** and **S10B Fig**), an AasS/C10 adenylate complex (PDB: 8JYU) was revealed to remain in an adenylate-forming conformation (**Fig 6E** and **6F**). Intriguingly, binding of C10-AMS inhibitor to apo-AasS (PDB: 8JYL) promoted a marked shift from its initial open conformation to the thioester-forming statue (**Fig 6G** and **6H**). Although that C10-AMS compound is a mimicry of C10-AMP adenylate (**Fig 2A** and **2B**), this inhibitor-induced conformation is unexpectedly analogous to the recently-reported complex structure of AasS/M-C7-ACP (**S10C** and **S10D Fig**) [58], rather than resembling that of C10 acyl adenylate (**S1** and **S10E** and **S10F** **Figs**). Structural alignment of C10-AMP adenylate-bound conformation with its C10-AMS inhibitor-liganded form, elucidated that conformational transition is due to the 140° rotation of the compact AasS_C domain (**S10G Fig**).

### Recognition of AasS by C10-AMS inhibitor

**Fig 7:**
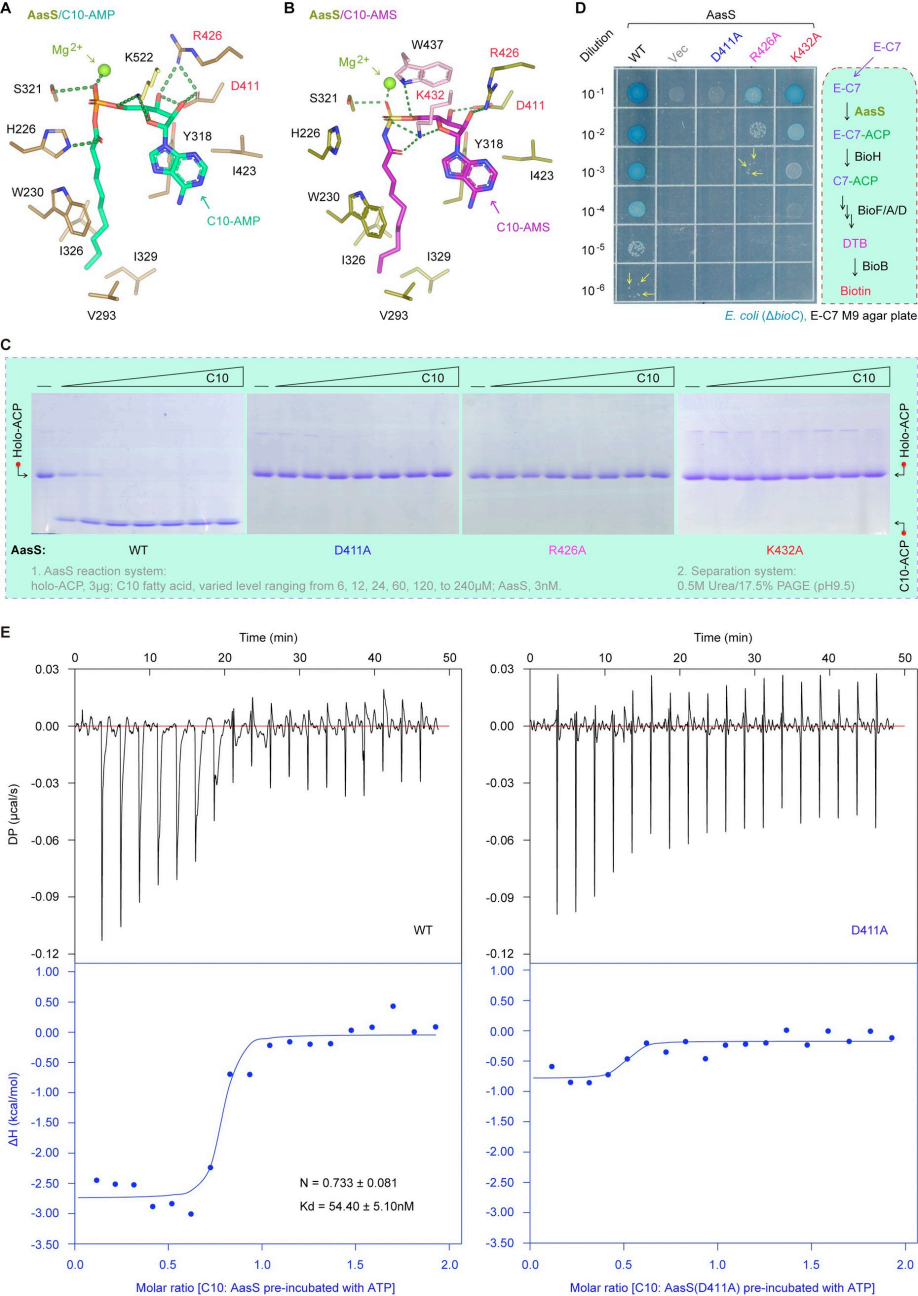
Parallels in binding of AasS to the C10-AMP adenylate intermediate and the C10-AMS inhibitor. Structural visualization for AasS binding an intermediate C10-AMP adenylate (**A**) and its inhibitor C10-AMS (**B**). Mg^2+^ atoms are displayed as spheres and residues closer to the position of ligands are shown in sticks form. New residues were seen as a result of the C-terminus rearrangement, as the pink sticks showed, with K432 being especially important. **C.** The three mutants of AasS (D411A, R426A, and K432A) are inactive with the C10 fatty acid substrate *in vitro*. Both AasS enzymatic reaction and conformationally-sensitive gel separation were conducted as described in **Fig 2D** and **2F**. **D.** *In vivo* evidence that the three single mutants of AasS (D411A, R426A, and K432A) lose the ability to bypass the physiological requirement of biotin for the *bioC* isogenic mutant of *E*. *coli* on the non-permissive condition. The bacterial viability on M9 defined agar medium was determined as performed in **Fig 4E**. **E.** ITC experiments revealed that WT of AasS binds to decanoic acid substrate, whereas the AasS (D411A) mutant does not. The ITC assays were carried out as we conducted in **Fig 3B**.

**Fig 8:**
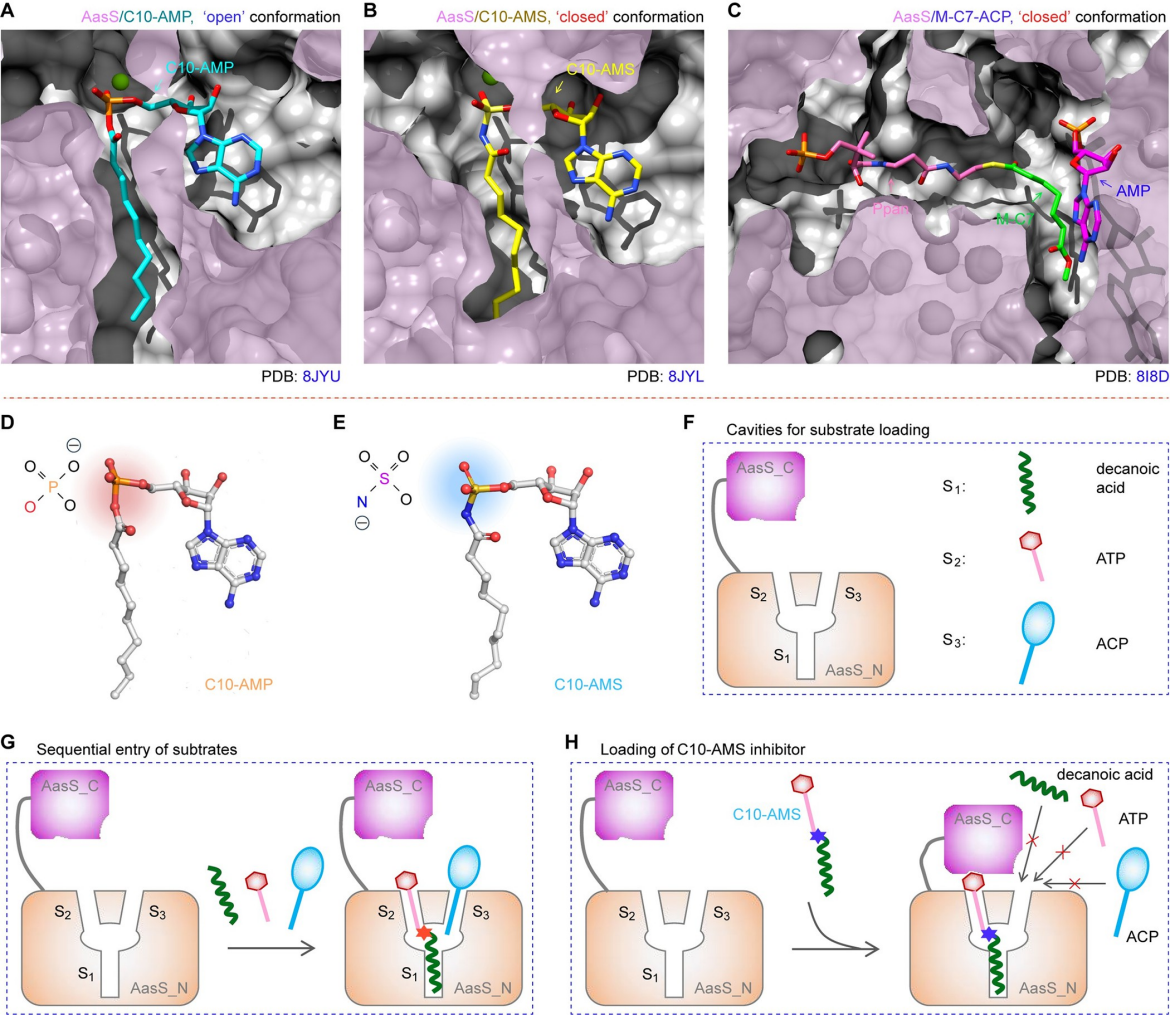
Schematic diagram for inhibition of AasS by the C10-AMS inhibitor. **A.** The cut-away view of a C10-AMP intermediate-loaded cavity of AasS enzyme (PDB: 8JYU). The C10-AMP molecule is shown in a stick representation and carbon atoms are colored in cyan. **B.** The cut-away view of AasS enzyme revealed that C10-AMS inhibitor occupies the C10-AMP intermediate-binding cavity (PDB: 8JYL). Like a C10-AMP molecule, the C10-AMS inhibitor is also given in a stick representation, of which carbon atoms are colored in yellow. **C.** The cut-away view of ACP cargo and released AMP in the substrate cavity of AasS/M-C7-ACP complex (PDB: 8I8D). The carbon atoms of Ppan arm, M-C7 and AMP are colored in pink, green, and magenta respectively. Chemical structures of C10-AMP intermediate (**D**) and C10-AMS inhibitor (**E**). It was given in ball-and-stick models. Red balls denoted oxygen atoms, blue balls referred to nitrogen atoms, and orange balls indicated phosphate atoms. Except for a sulfamoyl moiety that replaces a phosphate group, the C10-AMS inhibitor resembles the C10-AMP intermediate. **F.** Cartoon representation for open conformation of a two-subunit enzyme AasS, of which a large domain AasS_N contains a V-shape cavity for sequential loading of three distinct substrates (S_1_ to S_3_). Namely, they included decanoic acid (S_1_), ATP (S_2_), and ACP (S_3_). **G.** A working model for AasS in open conformation adapted to sequential binding its three unique substrates (S_1_ to S_3_). **H.** Scheme for C10-AMS inhibitor-induced closed formation of AasS that blocks the entry of three substrates (S_1_ to S_3_).

To explore how this inhibitor targets AasS enzyme, a panel of substrate/ligand-binding cavities were illustrated on the basis of three AasS structures. Apart from the C10-AMP intermediate-loading channel (**Figs 7A** and **8A**), its analog C10-AMS inhibitor-binding cavity (**Figs 7B** and **8B**) was proposed, along with the known tunnel injected by the M-C7 moiety of M-C7-ACP substrate (**Fig 8C**). As for the cavity for C10-AMP occupancy (**S11A Fig**), a C10-AMP molecule contacts several AasS_C active site (**Fig 7A**). In brief, an adenine base is held in place by the hydrophobic side chains of Y318 and I423, and the ribose hydroxyl groups form a hydrogen bond network with the two residues (D411 and R426). The K522 residue from the AasS_C domain interacts with the ribose ring oxygen atom and the bridging oxygen atom of AMP via two hydrogen bonds (**Fig 7A**). The AMP phosphate is coordinated by a Mg^2+^ atom as well as the hydroxyl groups of S321. The carbonyl group of C10-AMP gives a hydrogen bond with H226 site. The hydrophobic carbon chain of C10 is fixed in place by the four residues (V293, I326, I329, and the conserved W230). Notably, the W230 residue plays a gatekeeper role in eFA loading by AasS (**Fig 7A**), which is equivalent to the W234 of ttFACS [57].

As predicted, C10-AMS inhibitor (**S11B Fig**) was also found to bind an array of almost-identical active sites in the AasS_N part (**Fig 7B**). However, AasS_C domain adopts a closed conformation rather than an open state (**Fig 6**) to benefit its C10-AMS recognition. In place of K522 exploited in C10-AMP binding (**Fig 7A**), the AasS_C K432 residue produces several interactions with surrounding oxygen atoms (**Fig 7B**). Additionally, The W437 residue here forms a hydrogen bond with the bridging oxygen atom. Because of the bulky W437 side chain, it seemed likely that R426 rotates around 90 degrees to assure its interaction with the ribose. The interaction of C10-AMS with K432 residue promoted its carboxyl group rotation away from H226, rendering the basic residue H226 free. Given that the adenine moiety and carbon chain in the C10-AMS/AasS structure are coordinated in parallel to that of C10-AMP adenylate-bound structure (**Fig 7A** and **7B**), we selected three conserved and critical residues (D411, R426, and K432) for further structure-guided functional study. First, three single mutants of AasS enzymes with an alanine substitution (i.e., D411A, R426A, and K432A), were over-expressed and purified to homogeneity (**S12 Fig**). As shown in our gel filtration analysis, none of them is altered in the solution as a hexamer (**S12A Fig**). Next, the three AasS derivatives (D411A, R426A and K432A, in **S12B Fig**) were examined for the altered abilities of ligating C10 fatty acid to holo-ACP. As expected from our enzymatic assay with urea gel of conformational sensitivity, none of the three single mutations retains any detectable activity with C10 acyl substrate (**Fig 7C**). Moreover, any one mutation of the three residues greatly impairs the ability of AasS in biotin synthesis bypass *in vivo* (**Fig 7D**). The underlying mechanism can be due to the loss of function in substrate/ligand recognition, and this is largely verified by two lines of biophysical data. In agreement with the result of microscale thermophoresis (MST) (**S13A** and **S13B Fig**), ITC data showed that the mutant AasS (D411A) fails to effectively bind its cognate C10 acyl substrate (**Fig 7E**). In summary, the combination of enzymatic data *in vitro* and bacterial viability *in vivo*, constitutes a proof of concept for mixed inhibition of AasS by C10-AMS compound (**Fig 8**).

## Conclusions

The versatile AasS enzyme represents a druggable pathway of eFA scavenging exclusively encoded in over 13 *Vibrio* species except for *V*. *cholerae* [58]. Functional replacement with its two isoforms (*Neisseria* AasN [55] and *Chlamydia* AasC [33]) suggested an expansion of eFA salvage as a promising drug target. This postulate was recently demonstrated by an observation of C10-AMS as a broad inhibitor for acyl-ACP synthetases [60]. Given its potential relevance to clinical anti-bacterial therapies, we integrated cryo-EM, a cutting-edge approach to address the inhibitory mechanism of the lead compound C10-AMS on the basis of a paradigm AasS enzyme. The data reported here enabled us to provide mechanistic insights into functional impairment of eFA recycling by the mixed-type C10-AMS inhibitor via its efficient binding to AasS (**Fig 8D–8H**). It is likely that C10-AMS could bind to the ATP and fatty acid binding sites of the free enzyme as well as to the fatty acid binding site in the enzyme-ATP complex. Prior to this study, a relatively-complete structural landscape of AasS catalysis was illustrated, in which AasS is switched to a tetramer conformation from its dominant hexamer state by the ATP-Mg^2+^ cofactor [58]. Each protomer of AasS hexamer appears as a tropical pitcher plant composed of an N-terminal big body and a C-terminal compact cap (**Fig 8F**), of which the body is situated by substrate-binding sites (**Fig 8A–8C**). Presumably, the AasS monomer adopts an ‘open’ conformation to accept the sequential entry of various substrates ranging from decanoic acid, ATP, to holo-ACP (**Fig 8G**). Compared to the C10-AMP intermediate (**Fig 8D**), the C10-AMS inhibitor differs in its non-hydrolysable sulfamoyl linker (**Fig 8E**). Unexpectedly, this inhibitor was found to trap AasS enzyme in a ‘closed’ conformation (**Fig 8H**), which likely represents the enzyme at the very beginning of the second step reaction where the adenylate serves as a substrate, before the binding of the ACP thiol substrate. The resultant blockade prevents other substrates from binding/entering AasS enzyme (**Fig 8H**). Indeed, this working model we established was well supported by loss-of-function mutations of critical interface residues, dependent on our structure-guided alanine scanning mutagenesis (**Fig 7**).

We are aware that the ability of AasS to recycle environmental fatty acids facilitates *Vibrio* pathogens to circumvent FAS II inhibition [59,60]. As a dead-end substrate, C10-AMS compound renders AasS locked in a ‘closed’ conformation, and catalytically inactive. Consequently, this hampers eFA recycling/utilization via an AasS-ACP route. It is reasonable that the use of C10-AMS might re-sensitize bacterial pathogens to FAS II inhibitors (e.g., cerulenin). Additionally, it is plausible to ask the question of whether the other FakA/B system of eFA recycling can be targeted through specific inhibitors to reverse the emergence of FAS II bypass in some Gram-positive pathogens (**Fig 1**) [34–36,88]. In summary, our discovery provides a molecular basis for targeting of AasS by the C10-AMS inhibitor, and benefits the development of next-generation therapeutic, esp. the combination of FAS II inhibitors with C10-AMS scaffold-originated derivatives as adjuvants.

## Materials and methods

### Strains, plasmids, primers, and growth conditions

The bacterial strains included here were derivatives of *Escherichia coli* MG1655 (**S1 Table**). The BL21 (DE3) carrying pET28a::*aasS* was utilized to produce the recombinant AasS enzyme, and the Δ*bioC* mutant *E*. *coli*, a biotin auxotroph STL96 [84], was applied to analyze AasS-based bypass of BioC-directed initiating step of biotin bypass (**S1 Table**). Structure-guided, site-directed mutagenesis was employed to create three single mutants of *aasS* with a putative disruption in substrate/inhibitor binding (**S2 Table**). Namely, these mutants denoted D411A, R426A, and K432A. The constitutive expression vector of pET21a-P*rmpA* we recently developed [89], was adopted to examine *in vivo* role of AasS and its three single mutants in the context of biotin bypass (**S1 Table**) [58]. All the constructs were determined by direct DNA sequencing and multiplex PCR assays (**S2 Table**). Apart from Luria-Bertani (LB) broth, biotin-free M9 chemical defined medium was utilized for an AasS-aided assay of biotin bypass. To select certain plasmids, some antibiotics were added accordingly, including (i) ampicillin (100 μg/ml) for pET21a-P*rmpA* derivatives, and (ii) kanamycin (50 μg/ml) for pET28a-borne AasS and its mutants.

### Protein expression and purification

As described for the WT form of AasS [58], its three mutated versions, like AasS (D411A), were overexpressed and purified to homogeneity. In brief, cell pellets from 1 liter of cultures with pET28a-borne AasS expression were subjected to lysis with French press. The clarified lysates were incubated with Ni-NTA agarose (Qiagen) to capture the 6x his-tagged AasS proteins. After removal of contaminants, the AasS protein of interest was eluted with an elution buffer containing 150 mM imidazole, and then dialyzed against gel filtration buffer (25 mM Tris-HCl (pH 8.0), 150 mM NaCl and 2 mM DTT). Next, the AasS sample (~20 mg/ml), was subjected to the size exclusion chromatography (SEC) analysis on AKTA Pure, using a Superdex 200 Increase 10/300 column (GE Healthcare). The protein sample collected from the peak candidate was validated with SDS-PAGE (12%), which was followed by the *in vitro* enzymatic assays and cryo-EM studies, as well as enzymatic assays *in vitro*.

### *In vitro* enzymatic activity of AasS

As earlier established by Jiang and coworkers [47,48] with little alteration, we analyzed enzymatic activities of AasS and its mutants. In addition to the AasS addition, this reaction system consisted of 100 mM Tris-HCl (pH 7.5), 10 mM MgSO_4_, 5 mM DTT, and 10 mM ATP, fatty acyl substrate (~0.6 mM), and holo-ACP (~75 μg/ml). Two fatty acids tested here referred to mono-ethyl pimeloyl ester (E-C7/E-pim) and decanoic acid (C10). To ascertain its inhibitory effect on enzymatic activity, the varied level of C10-AMS inhibitor (BirdoTech, China) was pre-incubated with AasS. After 0.5 h of maintenance of AasS reaction at 37°C, the resultant acyl-ACP products were separated with a conformationally-sensitive 17.5% PAGE gel (pH9.5) containing 0.5 M urea [58]. Semi-quantitative curves were also plotted to determine how profound binding of C10-AMS inhibitor is relative to the substrate E-C7 and/or C10.

### Assays for bypass of AasS in biotin synthesis

Given that AasS ligates E-C7 fatty acid with holo-ACP, giving E-C7-ACP, an alternative precursor for bacterial biotin synthesis, plasmid-borne *aasS* expression in Δ*bioC* biotin auxotroph strain is assumed to overcome the requirement of the primary BioC step [58,83]. Apart from its WT, three single mutants of AasS (D411A, R426A, and K432A) were examined *in vivo*, of which the compromised activity is presumably proportional to the varied viability of the Δ*bioC* reporter strains on the non-permissive M9 growth condition. To test its inhibitory effect, the C10-AMS inhibitor in a series of dilution was supplemented [59].

### Isothermal titration calorimetry

The assays of Isothermal titration calorimetry (ITC) were conducted to address the stoichiometry of AasS binding to its C10-AMS inhibitor and C10-AMP intermediate. Since both malic acid and pimelic acid are not substrates for AasS, the two dicarboxylic acids were assumed as negative controls in our ITC analysis. Using a micro-calorimeter (MicroCal PEAQ-ITC) as recently described for E-C7 with little change [58], totally 18 titrations (2 μl each at an internal of 2.5 min) proceeded in the cell at room temperature. Prior to the titration, AasS protein (~40 μM) was saturated with its ATP cofactor (120 μM) in the cell, and C10-AMS inhibitor (or C10) was kept at 600 μM in the syringe. The titration buffer was composed of 150 mM NaCl, 25 mM Tris-HCl (pH8.0), 2 mM DTT, and 3 mM MgCl_2_. One set of site model by the MicroCal PEAQ-ITC software enabled plotting the titration curves. Next, the stoichiometry (N) and dissociation constant (Kd) were calculated. It was given as an average ± standard deviation (SD) from three independent trials.

### Microscale thermophoresis

To validate poor binding of AasS(D411A) to C10 fatty acyl adenylate observed in our ITC assays, the relatively-more sensitive approach of microscale thermophoresis (MST) was additionally applied here as Jerabek-Willemsen *et al*. [90] described with minor alteration. Using the Protein Labeling Kit (Protein Labeling Kit RED-NHS 2^nd^ Generation, Nano Temper Technology), both the wild-type AasS and its D411A mutant were fluorescently labelled in the MST buffer (150 mM NaCl, 25 mM Tris-HCl, 3 mM MgCl_2_, 0.05% Tween-20, pH 8.0). The ligand C10-AMP used in MST assays was prepared by an equal mixture of C10 and ATP. In general, an NT.115 Monolith instrument (Nano Temper Technology, Germany) was integrated, of which the routine parameters included a RED/LED (20%) excitation power along with 60% laser power for excitation. Then, the protein samples were siphoned into MST Premium Coated capillaries for the determination of binding affinity at room temperature. The final concentrations of different AasS samples were 40 nM for WT, and 180 nM for D411A, respectively. The mixture of C10/ATP was diluted in a gradient (from 0.15 nM to 5.0 μM). Three independent MST experiments were conducted here. The dissociation constants (Kd) were calculated using the software MO. Affinity Analysis v2.3. Of note, this was largely dependent on the K_D_ model with a 1:1 stoichiometry per binding partner.

### Cryo-EM grid preparation and data acquisition

Three kinds of cryo-EM grids were prepared, which separately corresponded to (i) AasS alone, (ii) AasS-liganded with C10-AMP intermediate, and (iii) complex of AasS with its inhibitor C10-AMS. As we recently established with little modification [58], the recombinant version of AasS purified (~20 mg/ml, 3 μl) was applied on glow-discharged holey carbon grids (Quantifoil Cu R1.2/1.3, 300 mesh). The resultant grids were routinely blotted for 4 s under 100% humidity at 4°C, followed by the plunge-freezing into liquid ethane using Vitrobot Mark IV (Thermo Fischer Scientific). As for apo-AasS image collection, the 300 kV Titan Krios microscope (Thermo Fischer Scientific) was operated with K2 detector (Gatan). To improve image resolution of AasS complexes with C10-AMP ligand (or C10-AMS inhibitor), the same microscope was equipped with Falcon 4 detector along with the additional Selectris energy filter (Thermo Fischer Scientific). Unlike it apo-form featuring the calibrated magnification (×29,000, a pixel size of 1.014 Å), the other two sets of AasS complexes were imaged at the magnification (×130,000), yielding a pixel size of 0.93 Å on images. The defocus range was set from −0.8 to −1.5 μm. Each micrograph was dose-fractionated to 40 frames under a dose rate of 8 e^−^ per pixel per second, with a total exposure time of 8 s for apo-form (or 6 s for its complexes), resulting in a total dose of ~64 e^−^ Å^−2^ for AasS alone, and 52 e^−^ Å^−2^ for the complexes. As for automatic data acquisition, a Serial EM software used for the apo-AasS version [91], and a smart EPU software [92] was applied for the two complexes.

### Image processing, 3D reconstructions, model building and refinement

Three sets of micrograph datasets we collected were subjected to automatic single particle cryo-EM analysis with RELION 4.0 [93], which were followed by the beam-induced motion correction with MotionCor2 [94]. Notably, Contrast Transfer Function (CTF) parameter were settled with Gctf v1.18 [95] to assure the validation of data correction. Next, AasS protein particles auto-picked using Gautomatch v0.56 (Kai Zhang, MRC Laboratory of Molecular Biology), were imported into cryoSAPRC3.3.1 [96] for 2D classification and two rounds of 3D classification. In general, a subset of qualified particles was imported back to RELION 4.0 [96], subjected to Bayesian polishing, and brought back to cryoSAPRC3.3.1 [96] for final reconstruction with Non-uniform refinement.

As expected, three sets of high-resolution cryo-EM maps were produced. In brief, (i) 2329 micrographs of AasS alone gave a total of 1,040,351 particles, of which the good subset (i.e., 334,713 particles) generated a final map at the global resolution of 2.53 Å (**S5 Fig**); (ii) 1,160,261 particles auto-picked from 3058 micrographs of C10-AMS-liganded AasS form, enabled us to build a good subset of 212,195 particles, leading to a high-resolution map at 2.33 Å (**S9 Fig**); and (iii) as for the AasS complexed with C10-AMP intermediate, 3339 micrographs we harvested, returned a total of 1,737,985 particles, of which 602,383 particles as a good subset allowed a map at the global resolution of 2.19 Å (**S7 Fig**).

Next, *de novo* atomic model of AasS alone was initially built in Coot [97], based on its 2.53 Å cryo-EM map. The resultant model of apo-formed AasS acted as a template enabled further solving the models of C10-AMP-bound AasS and its inhibitor-liganded form. Finally, structure models we obtained, were refined in PHENIX against cryo-EM maps for real-space refinement (**Table 1**), and all-atomically validated with the software of MolProbity [98].

## Supporting information

S1 Table Bacterial strains and plasmids used in this study. (DOCX)

S2 Table Primers used in this study. *The bold letters represent the codons dedicated to certain mutation, and the underlined letters denoted restriction sites.
(DOCX)

S1 Fig The order of C10-AMS addition affects its inhibitory efficacy on AasS activity. **A.** Cartoon model of two opposite conformational states for AasS catalysis. Unlike the “open” conformation of AasS liganded with C10 substrate or C10-AMP intermediate, a “closed” conformation was observed for AasS upon binding an inhibitor of C10-AMS. The small domain of AasS_C was indicated with a pearl-shape colored orange, and the large domain of AasS_N was given with a bitten apple colored purple. The three small molecules are shown in a model of sticks. Like the C10 substrate, C10-AMP intermediate was colored green. Whereas the C10-AMS inhibitor was highlighted in magenta. **B.** Use of conformationally-sensitive gel to assay altered activity of AasS with C10 fatty acid substrate, followed by the addition of C10-AMS inhibitor**. C.** Evaluation of decreased activity of AasS with C10 substrate, simultaneously incubated with the varied level of C10-AMS inhibitor**. D.** Visualization for an interfered C10 acylation of holo-ACP by the AasS enzyme pre-incubated with the C10-AMS inhibitor. **E.** Semi-quantitative curves demonstrated that the addition order of C10-AMS vs C10 substrate might affect the inhibitory efficacy. The enzymatic reaction system (50 μl) was consisted of (i) 3 nM of AasS, (ii) 3 μg of holo-ACP acceptor, and (iii) 80 μM of C10 substrate. The level of C10-AMS inhibitor was added in a series of 2-fold dilution (varying from 0.78 μM, 1.57 μM, …, to 25 mΜ, panels B-E). The 0.5 M urea/17.5% PAGE (pH9.5) was utilized to separate C10-acylated ACP from its acceptor holo-ACP (panels B-D). As described for E-C7 fatty acylation in **Fig 4C**, the ImageJ software was also applied to quantitate the relative activity (%) of C10 fatty acylation in AasS reaction. The graphs were plotted from three independent experiments, and the output was presented in average ± SD. Designations: the symbol “—” denotes no addition of C10-AMS inhibitor; The top triangle on right hand represents the addition of C10-AMS at varied level.
(TIF)

S2 Fig Use of Michaelis-Menten curves to evaluate inhibition mode of AasS by the C10-AMS molecule. Michaelis-Menten curves of AasS reaction for the ATP cofactor (**A**) and C10 acyl substrate (**C**), on the condition of C10-AMS inhibitor supplemented at varied concentrations. C10-AMS was added at different level (ranging from 0, 125, 250, to 500 nM). Based on the dogma of Michaelis-Menten equation, a number of crosspoints of two vertically-intersecting dashed-lines (indicated with arrows) were given to determine the values of Km and Vmax. Of note, the coordinate (x,y) of the resultant crosspoint denotes the number (Km, Vmax /2). The values of Km and Vmax for AasS inhibited by C10-AMS in relative to ATP cofactor (**B**) and C10 substrate (**D**). The decline tendency of both Km and Vmax suggested that C10-AMS inhibitor exerts effects on AasS action in the mixed mode. Designations: Km, Michaelis constant; Vmax, Maximum velocity.
(TIF)

S3 Fig ITC-based evidence that two dicarboxylic acids (pimelic acid and malic acid) cannot bind to the AasS enzyme. **A.** The ITC measurement revealed no binding of AasS to the dicarboxylic acid, pimelic acid. **B.** The ITC analysis for the other dicarboxylic acid of malic acid without an ability of binding the AasS enzyme. The inside chemical molecules separately refer to pimelic acid (panel A), and malic acid (panel B). Notably, unlike pimelic acid, malic acid is a dicarboxylic acid with an α-positional hydroxyl modification (colored magenta). Designations: ITC, Isothermal titration calorimetry; N, Stoichiometry; Kd, dissociation constant; DP, differential power; ΔH, enthalpy.
(TIF)

S4 Fig AasS cannot utilize the two dicarboxylic acids (pimelic acid and malic acid) in vitro and in vivo. **A.** In contrast to E-C7 substrate, the two dicarboxylic acids of pimelic acid and malic acid are inactive with AasS enzyme. The conformationally-sensitive gel of 0.5 M urea/17.5% PAGE (pH9.5) was applied to separate fatty acylated ACP from its acceptor holo-ACP (panel A). **B.** Failure of AasS-based biotin bypass for the biotin auxotroph Δ*bioC* when grown on the M9 defined medium with pimelic acid as sole carbon source. **C.** Expression of AasS cannot bypass biotin requirement of the biotin auxotroph Δ*bioC* on the condition of malic acid as sole carbon source. Three different fatty acids tested here include (i) mono-ethyl pimelic acid (E-C7), (ii) malic acid, and (iii) pimelic acid (panels B&C). The biotin-deficient M9 minimum medium that contained varied fatty acids as sole carbon source was applied to evaluate substrate specificity of AasS in the context of bacterial growth of the biotin auxotroph of *E*. *coli* Δ*bioC* strain.
(TIF)

S5 Fig Image-processing flowchart for apo-AasS. **A.** Cryo-EM images of apo-AasS protein. **B.** 2D classification of negatively-stained sample of AasS in apo-form**. C.** Image processing flowchart for the collection of apo-AasS data. **D.** The Fourier shell correlation (FSC) curve displays a final resolution of 2.53 Å for apo-AasS. **E.** Distribution of local resolution for apo-AasS density map in various views.
(TIF)

S6 Fig Overall structure of AasS enzyme in apo-form. **A.** Cryo-EM structure of apo-AasS hexamer in ribbon form. **B.** Surface structure of apo-AasS hexamer. The apo-form of AasS hexamer (150 x 140 x 80 Å) essentially acts as a trimer of dimers, of which monomeric unit (AasS/AasS’) is orderly numbered from I, II, …, to VI. The dimer interface was indicated with a red arrow, and the trimer interface was highlighted with a blue arrow. The top view (150 x 140 Å) was given in upper panel. Following the rotation of 90° counter-clockwise, its front view (150 x 80 Å) was presented in bottom panel. The AasS_C/AasS’-C domain was colored hot-pink or chartreuse, and the AasS_N/AasS’-N domain was displayed in purple or light-orange.
(TIF)

S7 Fig Image-processing flowchart for AasS bound by an intermediate C10-AMP. **A.** Cryo-EM images of protein AasS accompanied by C10-AMP adenylate. **B.** Selected class averages from 2D classification of C10-AMP-liganded AasS enzyme. **C.** Image processing flowchart for AasS liganded with C10-AMP intermediate. **D.** Fourier shell correlation (FSC) curve reveals a final resolution of 2.19 Å for AasS/C10-AMP complex. Notably, the gold standard FSC is equal to 0.143. **E.** Distribution of local resolution for the AasS/C10-AMP complex density map in various views.
(TIF)

S8 Fig Overall architecture of AasS liganded with an intermediate of C10-AMP adenylate. **A.** Ribbon structure of AasS accompanied by the C10-AMP intermediate. **B.** Surface presentation of AasS liganded with the C10-AMP adenylate. Regardless of binding to the ligand, C10-AMP adenylate, AasS constantly forms a hexamer, i.e., a trimer of dimers (150 x 140 x 80 Å). The monomeric unit AasS/AasS’ is sequentially numbered from I, II, …, to VI. Unlike the dimer interface that is indicated with a brown arrow, the trimer interface was shown with a blue arrow. The rotation of 90° counter-clockwise allowed the conversion of AasS from its top view (150 x 140 Å, in upper panel) to the front view (150 x 80 Å, in bottom panel). The AasS_C/AasS’_C domain was colored lime-green or grey, and the AasS_N/AasS’_N domain was displayed in cyan or hot-pink.
(TIF)

S9 Fig Image-processing flowchart for AasS liganded with the C10-AMS inhibitor. **A.** A representative of cryo-EM images from the protein AasS complexed with C10-AMS inhibitor. **B.** Selected class averages from 2D classification of AasS/C10-AMS complex. **C.** Image processing flowchart for the AasS/C10-AMS complex. **D.** Combined with the gold standard FSC of 0.143, the analysis of fourier shell correlation (FSC) curve enabled an assignment of AasS/C10-AMS inhibitor complex with a final resolution of 2.33 Å**. E.** Local resolution distribution of density map of AasS/C10-AMS complex in different views.
(TIF)

S10 Fig Structural comparison of various AasS complexes in distinct conformations. **A-B.** Structural alignment of C10-AMP adenylate-bound AasS with its apo-form. The AasS_C domain is colored pink or magenta, and both are in an ‘open’ orientation. **C-D.** Structural superposition of the C10-AMS inhibitor-liganded AasS with its substrate M-C7-ACP complex. The AasS_C domain is colored gold or yellow, and both are in a ‘closed’ orientation. The ACP is colored lime-green. **E-F.** Structural comparison of AasS/C10-AMS inhibitor to its intermediate adenylate complex. The AasS_C domain (in gold) arising from AasS/C10-AMS complex presents a ‘closed’ conformation. Whereas the counterpart (in magenta) of AasS/C10-AMP complex gives an ‘open’ conformation. **G.** Side-by-side view of the AasS_C domain of C10-AMS-bound AasS in comparison to its form liganded with an intermediate C10-ATP. The same β-strand and α-helix in the two AasS_C domains were highlighted in magenta to show the 140° rotation.
(TIF)

S11 Fig Cryo-EM densities for two ligands, C10-AMP adenylate and its analog C10-AMS. **A.** Cryo-EM density (blue mesh) for the C10-AMP adenylate from AasS complexed with its reaction intermediate (PDB: 8JYU, 2.3 Å). **B.** Cryo-EM density of the C10-AMS inhibitor bound by AasS enzyme (8JYL, 2.5 Å). The two well-resolved ligands (i.e., C10-AMP and C10-AMS) were contoured at 2σ, and displayed as sticks. Additionally, magnesium ions were shown as green spheres.
(TIF)

S12 Fig Purification and verification of the AasS enzyme and its three mutants. **A.** SEC analysis of AasS and three mutated versions. The SEC profile of AasS eluted at the position of ~11.73 ml suggested that all the three mutants retain the solution structure of hexamer. In addition to WT, the three AasS mutants included D411A, R426A, and K432A, respectively. **B.** SDS-PAGE (12%) profile of the purified AasS derivatives. The AasS protein of ~65 kDa was highlighted with an arrow. Abbreviations: SEC, Size exclusion chromatography; kDa, kilo-Dalton; M, protein marker.
(TIF)

S13 Fig MST-based evidence that the AasS(D411A) mutant cannot bind to C10 fatty acyl substrate. **A.** MST assays suggested efficient binding of C10 acyl adenylate by the wild-type AasS enzyme. **B.** The alanine substitution of D411A rendered AasS enzyme to lose its ability of binding C10 acyl adenylate in the MST experiments. The data was presented in mean ± SD (n, 3 independent trials). The data generally agreed with the conclusion by our ITC analyses. Abbreviations: MST, Microscale thermophoresis; ΔF_norm_, the difference in normalized fluorescence against the concentration of its non-fluorescent ligand molecule.
(TIF)

S1 Data Source data for Figs 2E, 2G, 3B, 3C, 4C and 7E. (XLSX)

S2 Data Source data for S1E, S2A, S2C, S3A, S3B, S12A, S13A and S13B Figs. (XLSX)
