# Supplementary material for: An inhibitory mechanism of AasS, an exogenous fatty acid scavenger: Implications for re-sensitization of FAS II antimicrobials
Source: PLoS Pathog. 2024 Jul 15;20(7):e1012376. doi: 10.1371/journal.ppat.1012376 (PMC11271967; doi:10.1371/journal.ppat.1012376)
Supplement: S1 Table — (DOCX) [file ppat.1012376.s001.docx]

**S1 Table** Bacterial strains and plasmids used in this study

| Strains/plasmids | Description | Origins |
| --- | --- | --- |
| Strains | | |
| DH5α | A cloning host of *E. coli* | Lab stock |
| STL96 | The *bioC* deletion mutant of *E*. *coli* MG1655 strain (Δ*bioC*) | Lab stock |
| FYJ540 | DK574, a holo-ACP producing strain | Lab stock |
| FYJ582 | BL21(DE3) carrying pET28a*::aasS* | Lab stock |
| FYJ6001 | STL96(MG1655, Δ*bioC*) carrying pET21a-P*rmpA::aasS* | Lab stock |
| FYJ1229 | STL96(MG1655, Δ*bioC*) carrying pET21a-P*rmpA::aasS*(D411A) | This work |
| FYJ1230 | STL96(MG1655, Δ*bioC*) carrying pET21a-P*rmpA::aasS*(D426A) | This work |
| FYJ1231 | STL96(MG1655, Δ*bioC*) carrying pET21a-P*rmpA::aasS*(K432A) | This work |
| FYJ1232 | BL21(DE3) carrying  pET28a*::aasS*(D411A) | This work |
| FYJ1233 | BL21(DE3) carrying  pET28a*::aasS*(R426A) | This work |
| FYJ1234 | BL21(DE3) carrying pET28a*::aasS*(K432A) | This work |
| Plasmids | | |
| pET28a | The T7 promoter-driven expression vector, Km^R^ | Novagen |
| pET28a*::aasS* | pET28a carrying *aasS,* Km^R^ | Lab stock |
| pET28a*::aasS* (D411A) | pET28a carrying the *aasS*(D411A) mutant, Km^R^ | This work |
| pET28a*::aasS*(R426A) | pET28a carrying the *aasS*(R426A) mutant, Km^R^ | This work |
| pET28a*::aasS*(K432A) | pET28a carrying the *aasS*(K432A) mutant, Km^R^ | This work |
| pET21a-P*rmpA* | The *rmpA* promoter-driven expression vector, AmpR | Lab stock |
| pET21a-P*rmpA::aasS* | The pET21a-P*rmpA* vector carrying *aasS,* Amp^R^ | Lab stock |
| pET21a-P*rmpA::aasS*(D411A) | The pET21a-P*rmpA* vector encoding the *aasS*(D411A) mutant, Amp^R^ | This work |
| pET21a-P*rmpA::aasS*(R426A) | The pET21a-P*rmpA* vector expressing the *aasS*(R426A) mutant, Amp^R^ | This work |
| pET21a-P*rmpA::aasS*(K432A) | The pET21a-P*rmpA* vector encoding the *aasS*(K432A) mutant, Amp^R^ | This work |
