# Supplementary material for: An inhibitory mechanism of AasS, an exogenous fatty acid scavenger: Implications for re-sensitization of FAS II antimicrobials
Source: PLoS Pathog. 2024 Jul 15;20(7):e1012376. doi: 10.1371/journal.ppat.1012376 (PMC11271967; doi:10.1371/journal.ppat.1012376)
Supplement: S2 Table — *The bold letters represent the codons dedicated to certain mutation, and the underlined letters denoted restriction sites. (DOCX) [file ppat.1012376.s002.docx]

**S2** **Table** Primers used in this study

| Primers | Sequences * |
| --- | --- |
| AasS(D411A)-F | 5’-GT**G CA**G TGG CGC ATA TTG ATG ACG AAG GCT TT-3’ |
| AasS(D411A)-R | 5’-AAT ATG CGC CAC **TGC** ACC TGT GTG CAG GTA ACC GC-3’ |
| AasS(R426A)-F | 5’-CAC TGA C**GC A**GT AAA AGA TAT GAT TAA GAT ATC TGG TGA GTG-3’ |
| AasS(R426A)-R | 5’-CTT TTA C**TG C**GT CAG TGA TCT TGA TAA AGC CTT CG-3’ |
| AasS(K432A)-F | 5'-**GCA** ATA TCT GGT GAG TGG GTA AGC TCT TTA GA-3' |
| AasS(K432A)-R | 5'-CAC TCA CCA GAT AT**T GC**A ATC ATA TCT TTT ACG CGG TCA GTG-3' |
| pET21a-P*rmpA::aasS*-F(NdeI) | 5'-CAA AGT TAC TGT TTC TCA TAT GAA CCA GTA TGT AAA TGA TCC-3' |
| pET21a-P*rmpA::aasS*-R(XhoI) | 5'-GTG GTG GTG GTG GTG GTG CTC GAG CAG ATG AAG TTT ACG CAG TTC T-3' |
| pET28a*::aasS*-F(BamHI) | 5'-CGG GAT CCA TGA ACC AGT ATG TAA ATG ATC-3' |
| pET28a*::aasS*-R(XhoI) | 5'-CCG CTC GAG TTA CAG ATG AAG TTT ACG CAG TTC-3' |

*The bold letters represent the codons dedicated to certain mutation, and the underlined letters denoted restriction sites.
