# Supplementary figures and images for: An inhibitory mechanism of AasS, an exogenous fatty acid scavenger: Implications for re-sensitization of FAS II antimicrobials
Source: PLoS Pathog. 2024 Jul 15;20(7):e1012376. doi: 10.1371/journal.ppat.1012376 (PMC11271967; doi:10.1371/journal.ppat.1012376)

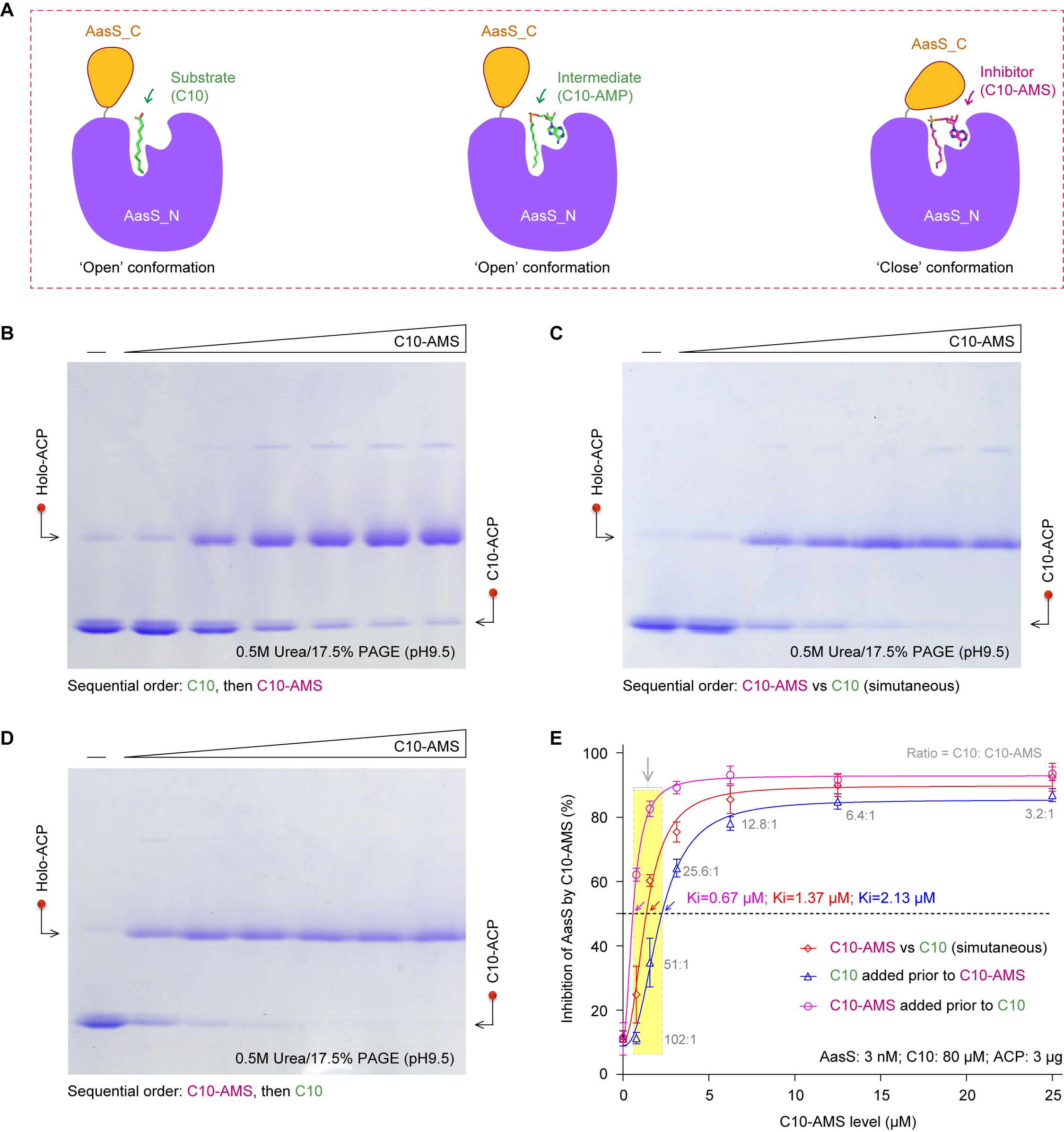

Supplement: S1 Fig — A. Cartoon model of two opposite conformational states for AasS catalysis. Unlike the “open” conformation of AasS liganded with C10 substrate or C10-AMP intermediate, a “closed” conformation was observed for AasS upon binding an inhibitor of C10-AMS. The small domain of AasS_C was indicated with a pearl-shape colored orange, and the large domain of AasS_N was given with a bitten apple colored purple. The three small molecules are shown in a model of sticks. Like the C10 substrate, C10-AMP intermediate was colored green. Whereas the C10-AMS inhibitor was highlighted in magenta. B. Use of conformationally-sensitive gel to assay altered activity of AasS with C10 fatty acid substrate, followed by the addition of C10-AMS inhibitor. C. Evaluation of decreased activity of AasS with C10 substrate, simultaneously incubated with the varied level of C10-AMS inhibitor. D. Visualization for an interfered C10 acylation of holo-ACP by the AasS enzyme pre-incubated with the C10-AMS inhibitor. E. Semi-quantitative curves demonstrated that the addition order of C10-AMS vs C10 substrate might affect the inhibitory efficacy. The enzymatic reaction system (50 μl) was consisted of (i) 3 nM of AasS, (ii) 3 μg of holo-ACP acceptor, and (iii) 80 μM of C10 substrate. The level of C10-AMS inhibitor was added in a series of 2-fold dilution (varying from 0.78 μM, 1.57 μM, …, to 25 mΜ, panels B-E). The 0.5 M urea/17.5% PAGE (pH9.5) was utilized to separate C10-acylated ACP from its acceptor holo-ACP (panels B-D). As described for E-C7 fatty acylation in Fig 4C, the ImageJ software was also applied to quantitate the relative activity (%) of C10 fatty acylation in AasS reaction. The graphs were plotted from three independent experiments, and the output was presented in average ± SD. Designations: the symbol “—” denotes no addition of C10-AMS inhibitor; The top triangle on right hand represents the addition of C10-AMS at varied level. (TIF) [file ppat.1012376.s003.tif]

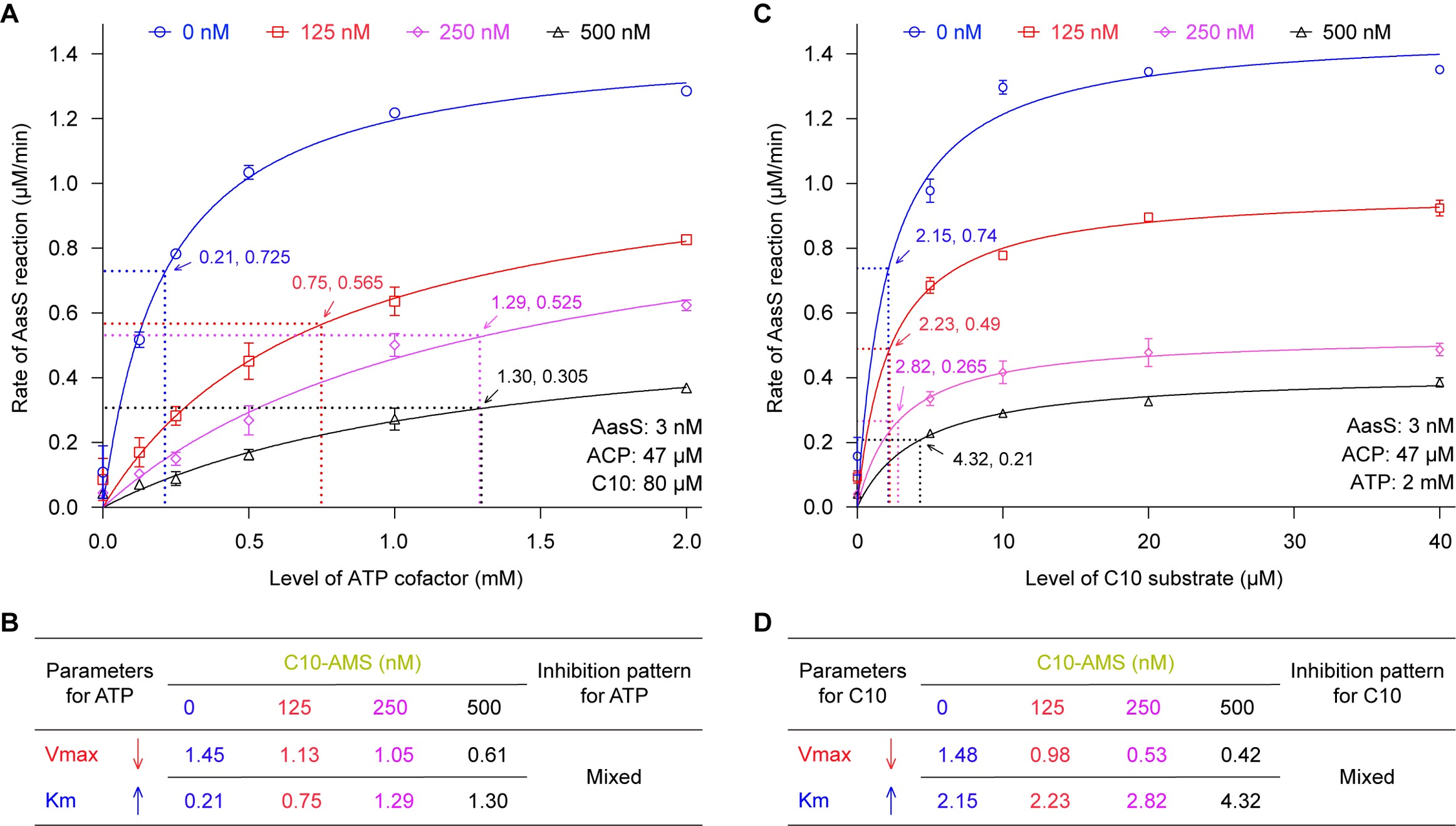

Supplement: S2 Fig — Michaelis-Menten curves of AasS reaction for the ATP cofactor (A) and C10 acyl substrate (C), on the condition of C10-AMS inhibitor supplemented at varied concentrations. C10-AMS was added at different level (ranging from 0, 125, 250, to 500 nM). Based on the dogma of Michaelis-Menten equation, a number of crosspoints of two vertically-intersecting dashed-lines (indicated with arrows) were given to determine the values of Km and Vmax. Of note, the coordinate (x,y) of the resultant crosspoint denotes the number (Km, Vmax /2). The values of Km and Vmax for AasS inhibited by C10-AMS in relative to ATP cofactor (B) and C10 substrate (D). The decline tendency of both Km and Vmax suggested that C10-AMS inhibitor exerts effects on AasS action in the mixed mode. Designations: Km, Michaelis constant; Vmax, Maximum velocity. (TIF) [file ppat.1012376.s004.tif]

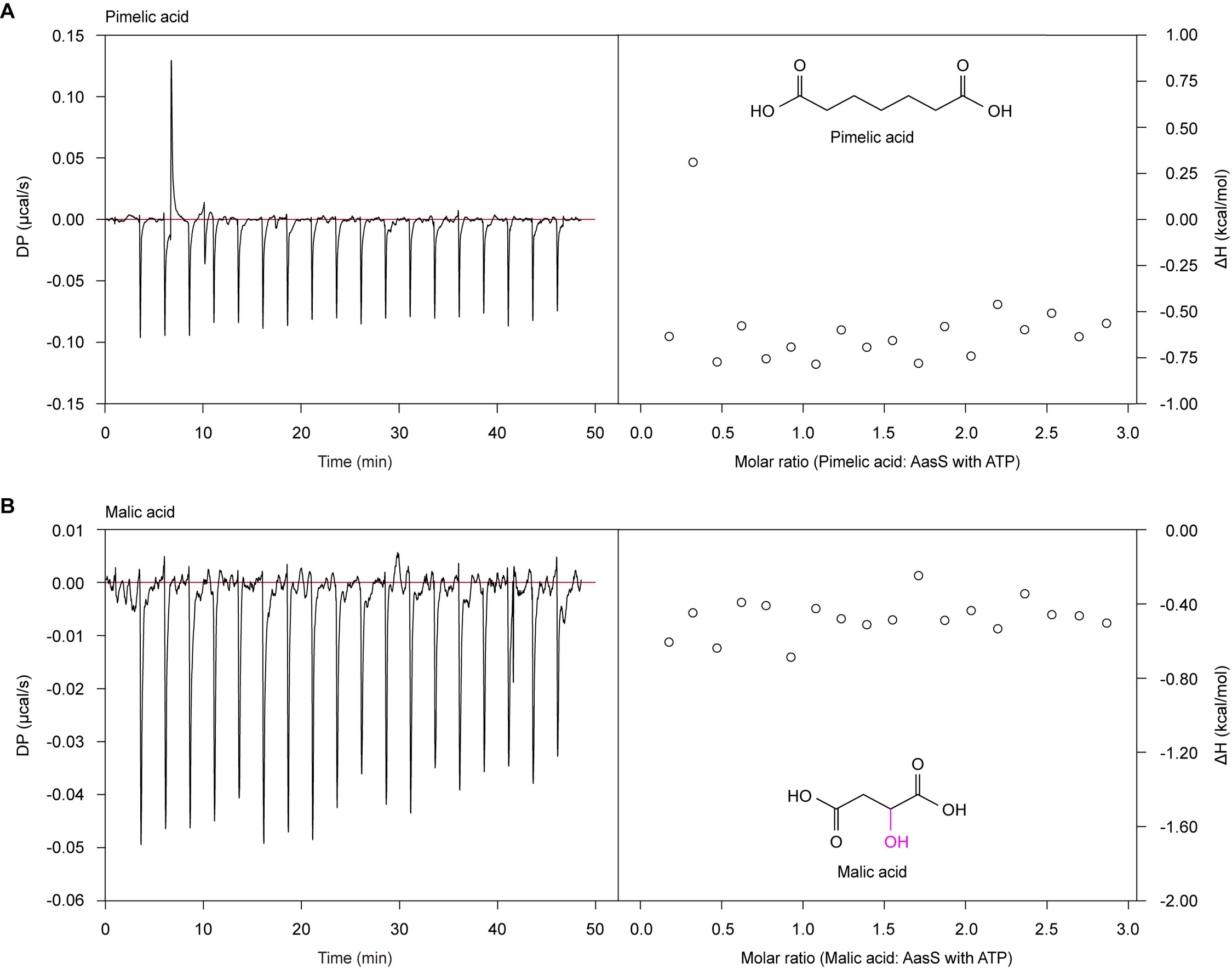

Supplement: S3 Fig — A. The ITC measurement revealed no binding of AasS to the dicarboxylic acid, pimelic acid. B. The ITC analysis for the other dicarboxylic acid of malic acid without an ability of binding the AasS enzyme. The inside chemical molecules separately refer to pimelic acid (panel A), and malic acid (panel B). Notably, unlike pimelic acid, malic acid is a dicarboxylic acid with an α-positional hydroxyl modification (colored magenta). Designations: ITC, Isothermal titration calorimetry; N, Stoichiometry; Kd, dissociation constant; DP, differential power; ΔH, enthalpy. (TIF) [file ppat.1012376.s005.tif]

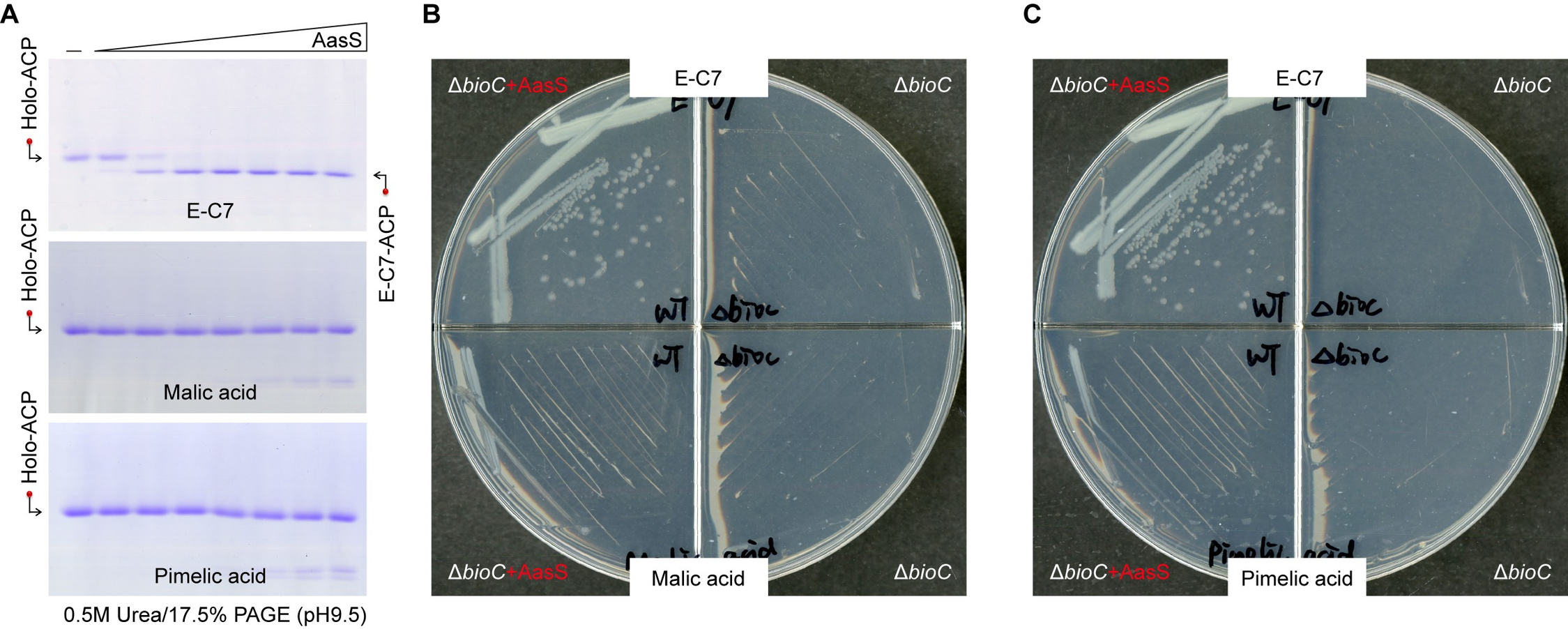

Supplement: S4 Fig — A. In contrast to E-C7 substrate, the two dicarboxylic acids of pimelic acid and malic acid are inactive with AasS enzyme. The conformationally-sensitive gel of 0.5 M urea/17.5% PAGE (pH9.5) was applied to separate fatty acylated ACP from its acceptor holo-ACP (panel A). B. Failure of AasS-based biotin bypass for the biotin auxotroph ΔbioC when grown on the M9 defined medium with pimelic acid as sole carbon source. C. Expression of AasS cannot bypass biotin requirement of the biotin auxotroph ΔbioC on the condition of malic acid as sole carbon source. Three different fatty acids tested here include (i) mono-ethyl pimelic acid (E-C7), (ii) malic acid, and (iii) pimelic acid (panels B&C). The biotin-deficient M9 minimum medium that contained varied fatty acids as sole carbon source was applied to evaluate substrate specificity of AasS in the context of bacterial growth of the biotin auxotroph of E. coli ΔbioC strain. (TIF) [file ppat.1012376.s006.tif]

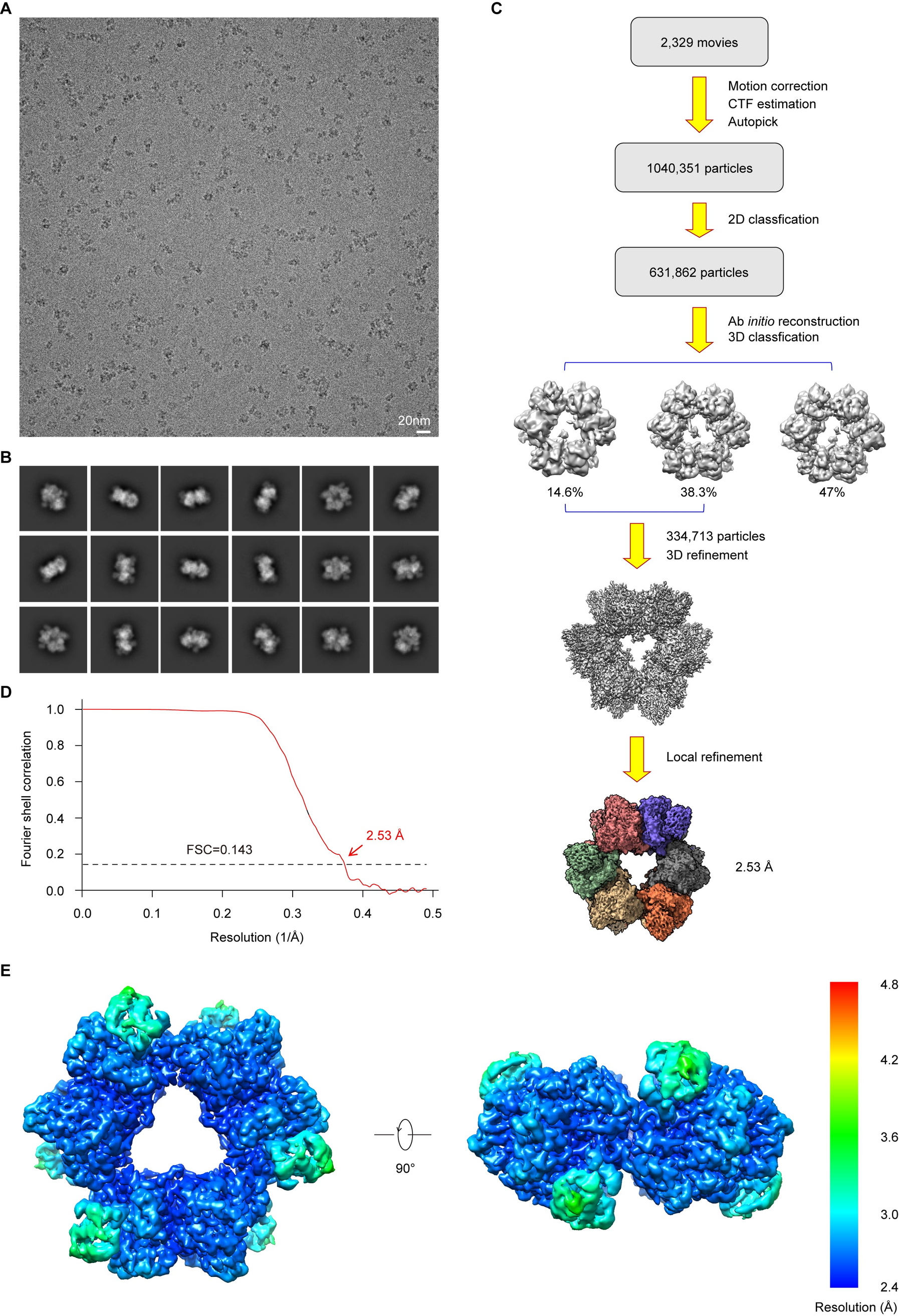

Supplement: S5 Fig — A. Cryo-EM images of apo-AasS protein. B. 2D classification of negatively-stained sample of AasS in apo-form. C. Image processing flowchart for the collection of apo-AasS data. D. The Fourier shell correlation (FSC) curve displays a final resolution of 2.53 Å for apo-AasS. E. Distribution of local resolution for apo-AasS density map in various views. (TIF) [file ppat.1012376.s007.tif]

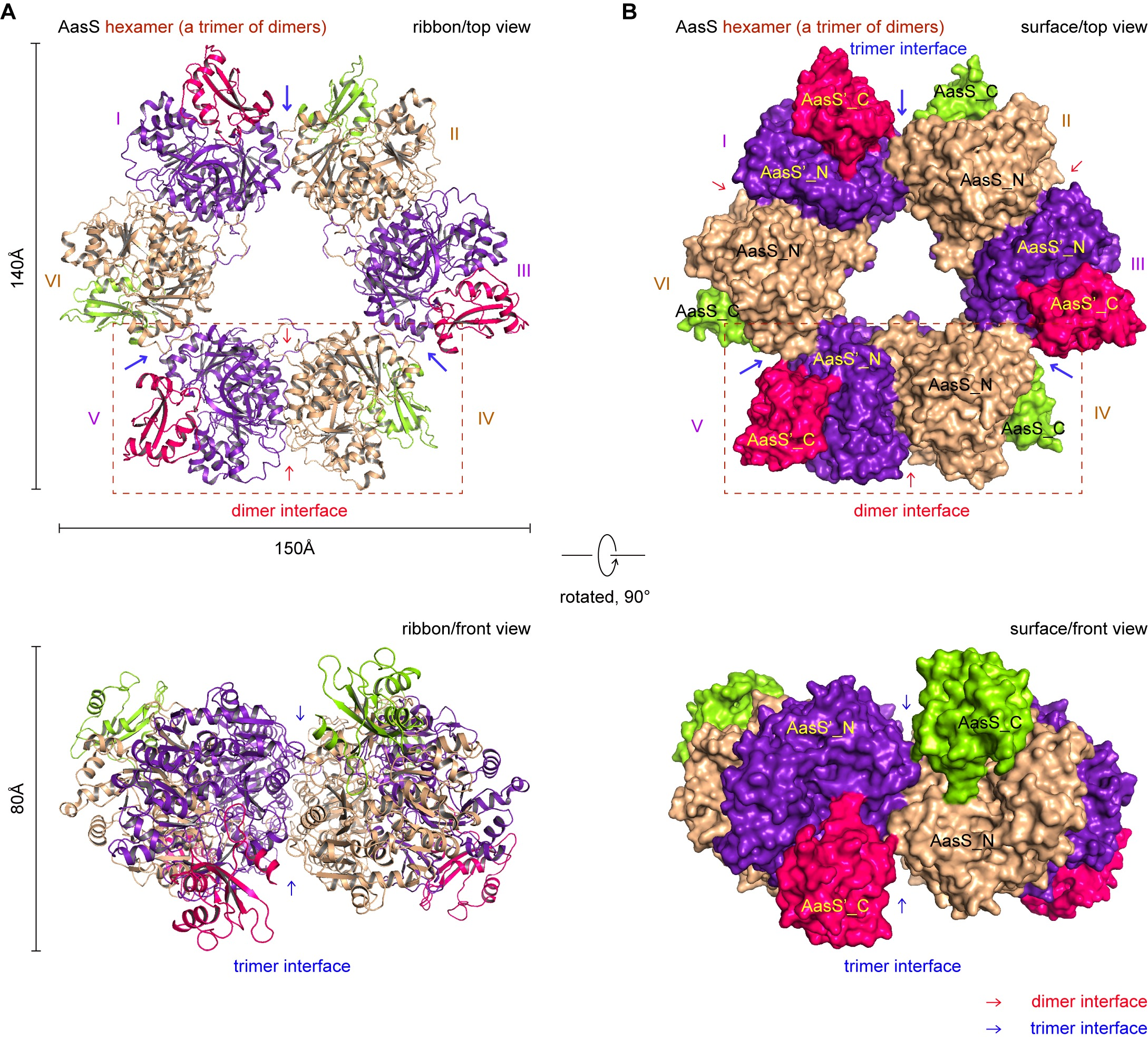

Supplement: S6 Fig — A. Cryo-EM structure of apo-AasS hexamer in ribbon form. B. Surface structure of apo-AasS hexamer. The apo-form of AasS hexamer (150 x 140 x 80 Å) essentially acts as a trimer of dimers, of which monomeric unit (AasS/AasS’) is orderly numbered from I, II, …, to VI. The dimer interface was indicated with a red arrow, and the trimer interface was highlighted with a blue arrow. The top view (150 x 140 Å) was given in upper panel. Following the rotation of 90° counter-clockwise, its front view (150 x 80 Å) was presented in bottom panel. The AasS_C/AasS’-C domain was colored hot-pink or chartreuse, and the AasS_N/AasS’-N domain was displayed in purple or light-orange. (TIF) [file ppat.1012376.s008.tif]

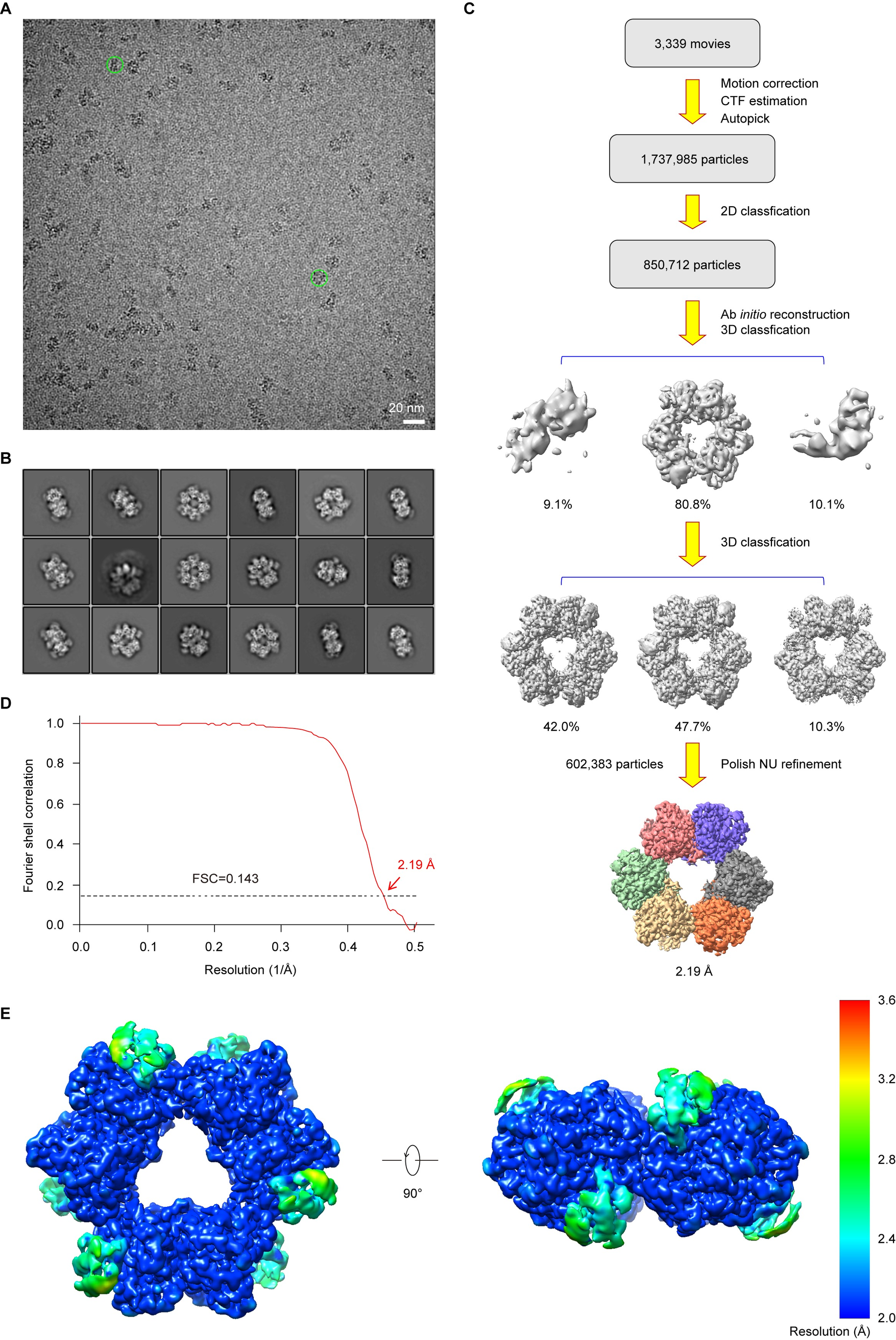

Supplement: S7 Fig — A. Cryo-EM images of protein AasS accompanied by C10-AMP adenylate. B. Selected class averages from 2D classification of C10-AMP-liganded AasS enzyme. C. Image processing flowchart for AasS liganded with C10-AMP intermediate. D. Fourier shell correlation (FSC) curve reveals a final resolution of 2.19 Å for AasS/C10-AMP complex. Notably, the gold standard FSC is equal to 0.143. E. Distribution of local resolution for the AasS/C10-AMP complex density map in various views. (TIF) [file ppat.1012376.s009.tif]

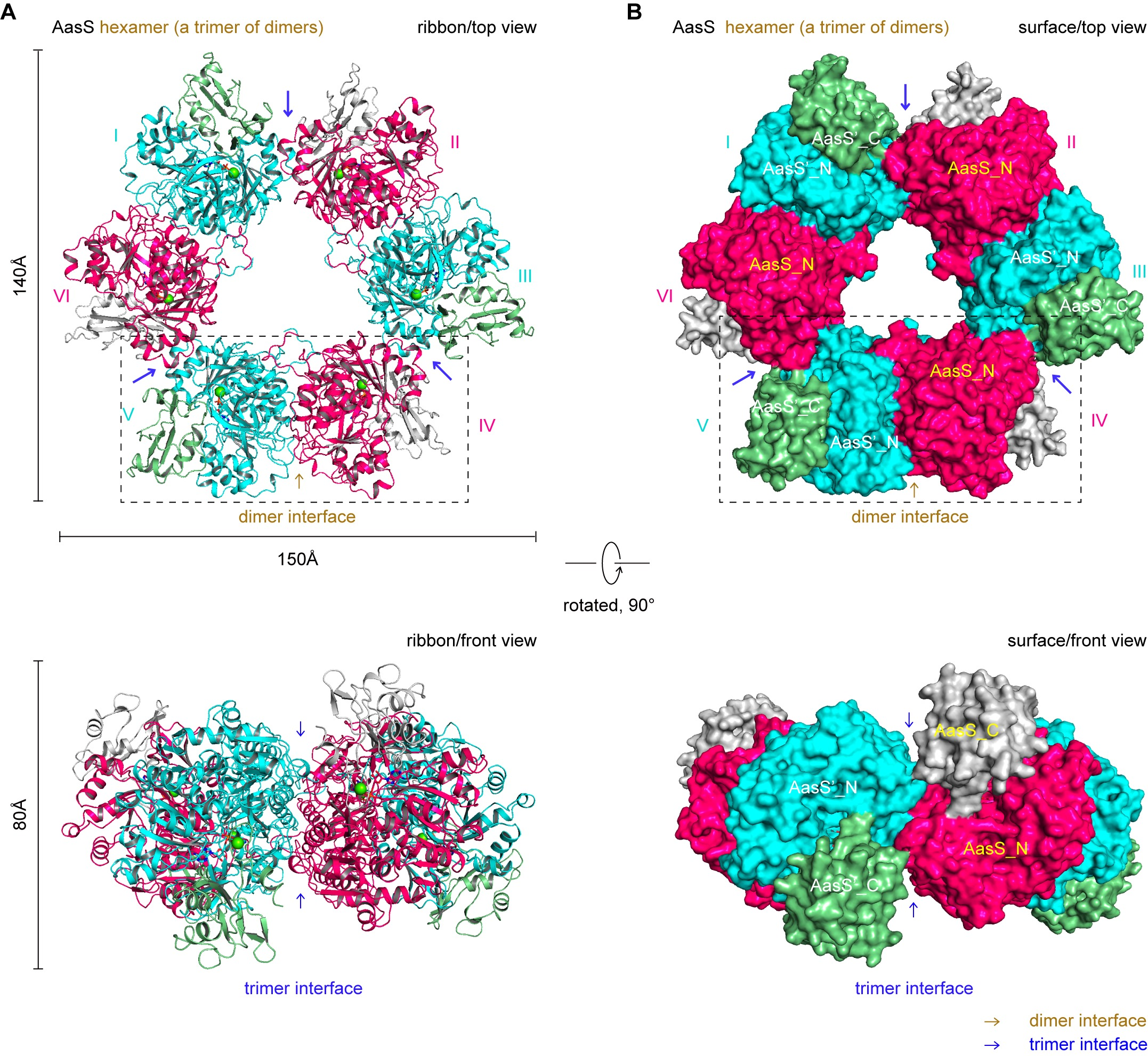

Supplement: S8 Fig — A. Ribbon structure of AasS accompanied by the C10-AMP intermediate. B. Surface presentation of AasS liganded with the C10-AMP adenylate. Regardless of binding to the ligand, C10-AMP adenylate, AasS constantly forms a hexamer, i.e., a trimer of dimers (150 x 140 x 80 Å). The monomeric unit AasS/AasS’ is sequentially numbered from I, II, …, to VI. Unlike the dimer interface that is indicated with a brown arrow, the trimer interface was shown with a blue arrow. The rotation of 90° counter-clockwise allowed the conversion of AasS from its top view (150 x 140 Å, in upper panel) to the front view (150 x 80 Å, in bottom panel). The AasS_C/AasS’_C domain was colored lime-green or grey, and the AasS_N/AasS’_N domain was displayed in cyan or hot-pink. (TIF) [file ppat.1012376.s010.tif]

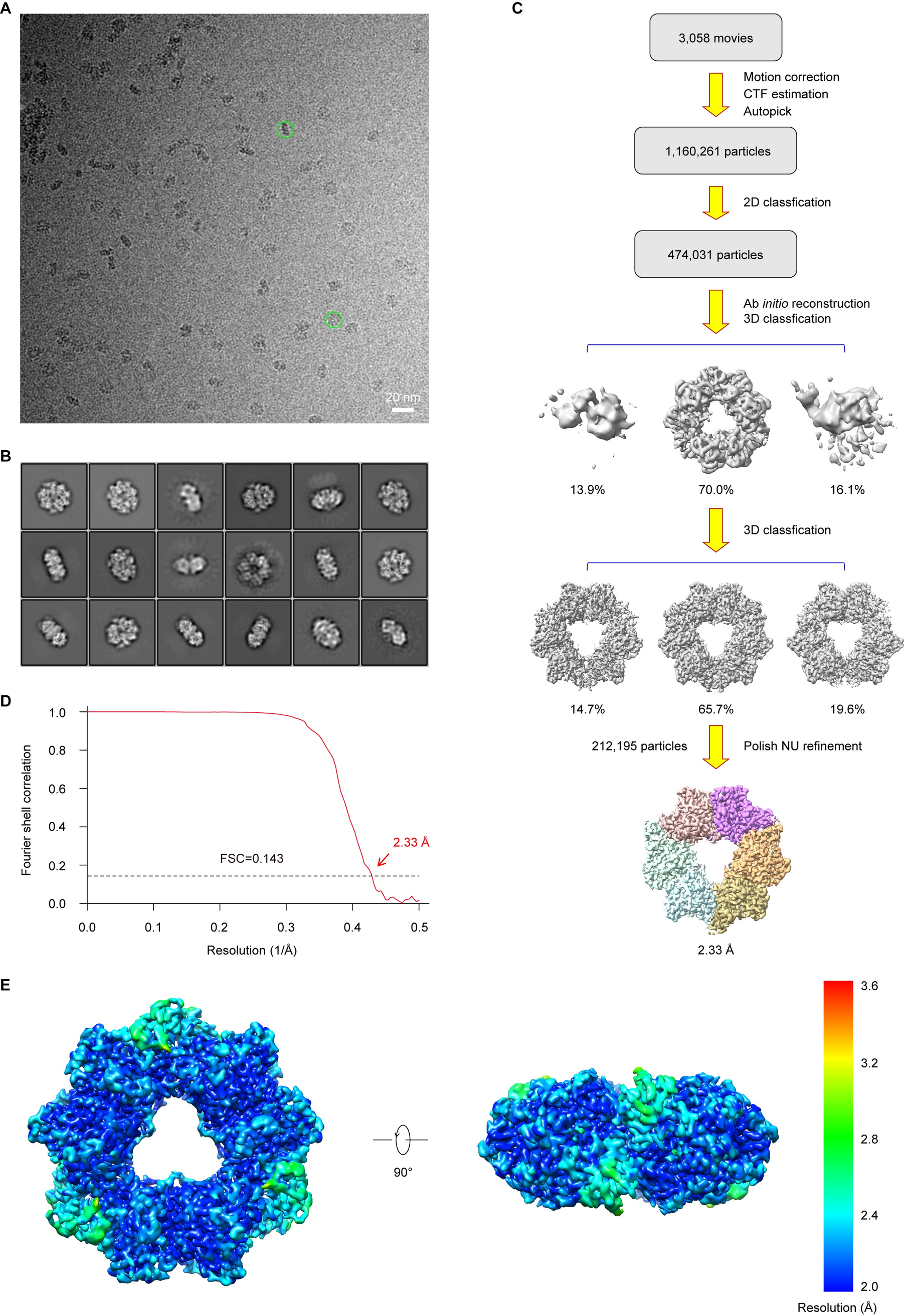

Supplement: S9 Fig — A. A representative of cryo-EM images from the protein AasS complexed with C10-AMS inhibitor. B. Selected class averages from 2D classification of AasS/C10-AMS complex. C. Image processing flowchart for the AasS/C10-AMS complex. D. Combined with the gold standard FSC of 0.143, the analysis of fourier shell correlation (FSC) curve enabled an assignment of AasS/C10-AMS inhibitor complex with a final resolution of 2.33 Å. E. Local resolution distribution of density map of AasS/C10-AMS complex in different views. (TIF) [file ppat.1012376.s011.tif]

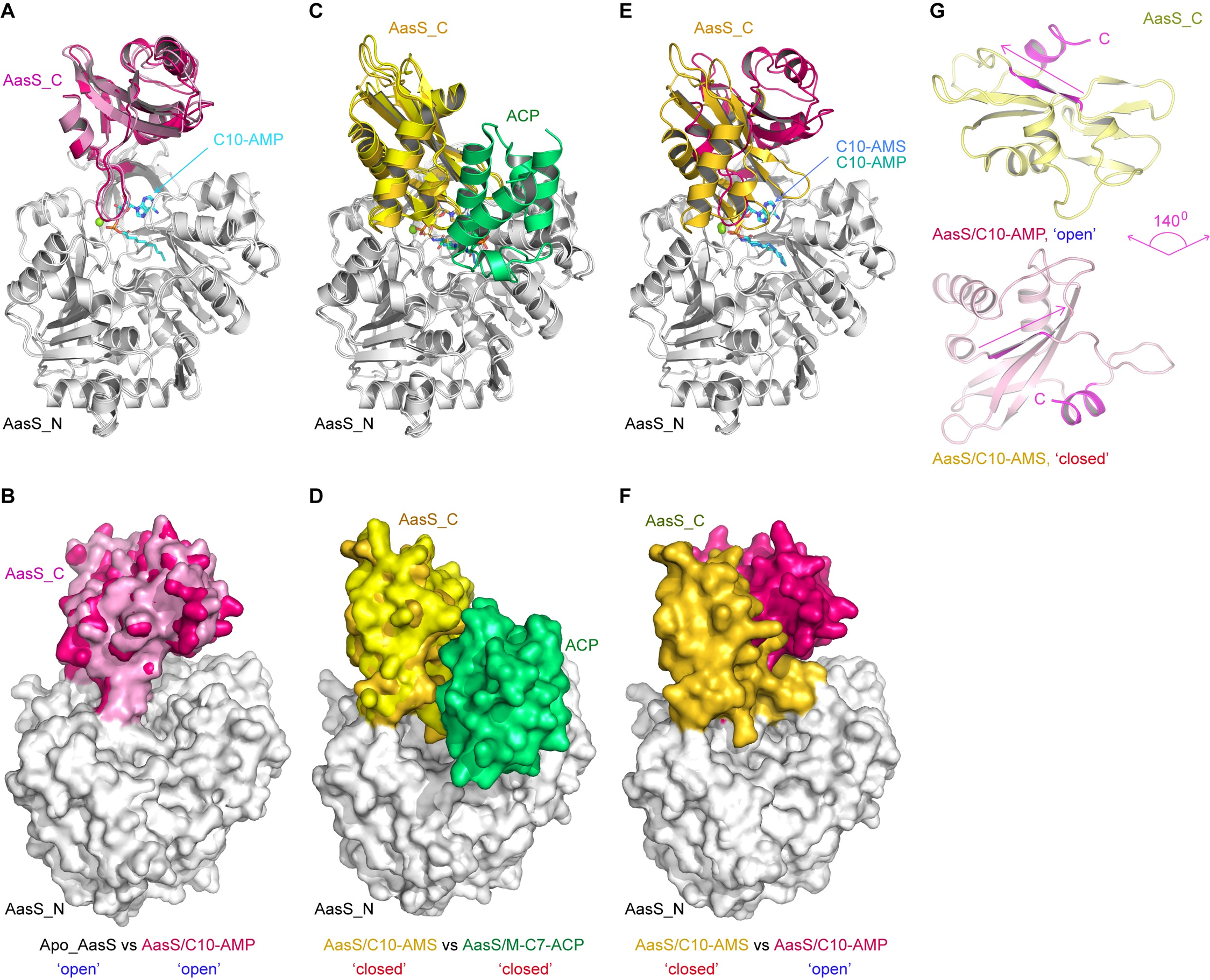

Supplement: S10 Fig — A-B. Structural alignment of C10-AMP adenylate-bound AasS with its apo-form. The AasS_C domain is colored pink or magenta, and both are in an ‘open’ orientation. C-D. Structural superposition of the C10-AMS inhibitor-liganded AasS with its substrate M-C7-ACP complex. The AasS_C domain is colored gold or yellow, and both are in a ‘closed’ orientation. The ACP is colored lime-green. E-F. Structural comparison of AasS/C10-AMS inhibitor to its intermediate adenylate complex. The AasS_C domain (in gold) arising from AasS/C10-AMS complex presents a ‘closed’ conformation. Whereas the counterpart (in magenta) of AasS/C10-AMP complex gives an ‘open’ conformation. G. Side-by-side view of the AasS_C domain of C10-AMS-bound AasS in comparison to its form liganded with an intermediate C10-ATP. The same β-strand and α-helix in the two AasS_C domains were highlighted in magenta to show the 140° rotation. (TIF) [file ppat.1012376.s012.tif]

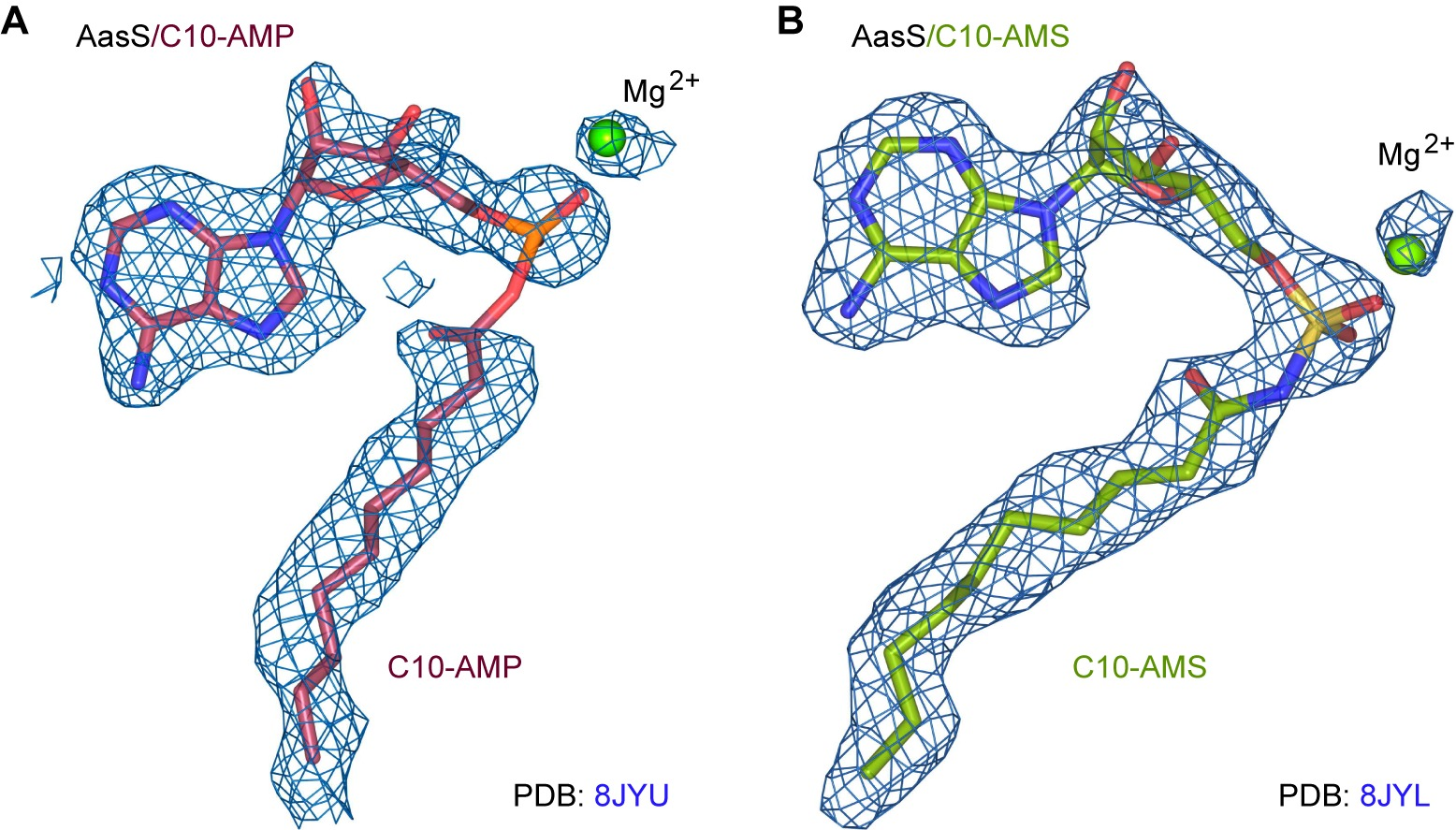

Supplement: S11 Fig — A. Cryo-EM density (blue mesh) for the C10-AMP adenylate from AasS complexed with its reaction intermediate (PDB: 8JYU, 2.3 Å). B. Cryo-EM density of the C10-AMS inhibitor bound by AasS enzyme (8JYL, 2.5 Å). The two well-resolved ligands (i.e., C10-AMP and C10-AMS) were contoured at 2σ, and displayed as sticks. Additionally, magnesium ions were shown as green spheres. (TIF) [file ppat.1012376.s013.tif]

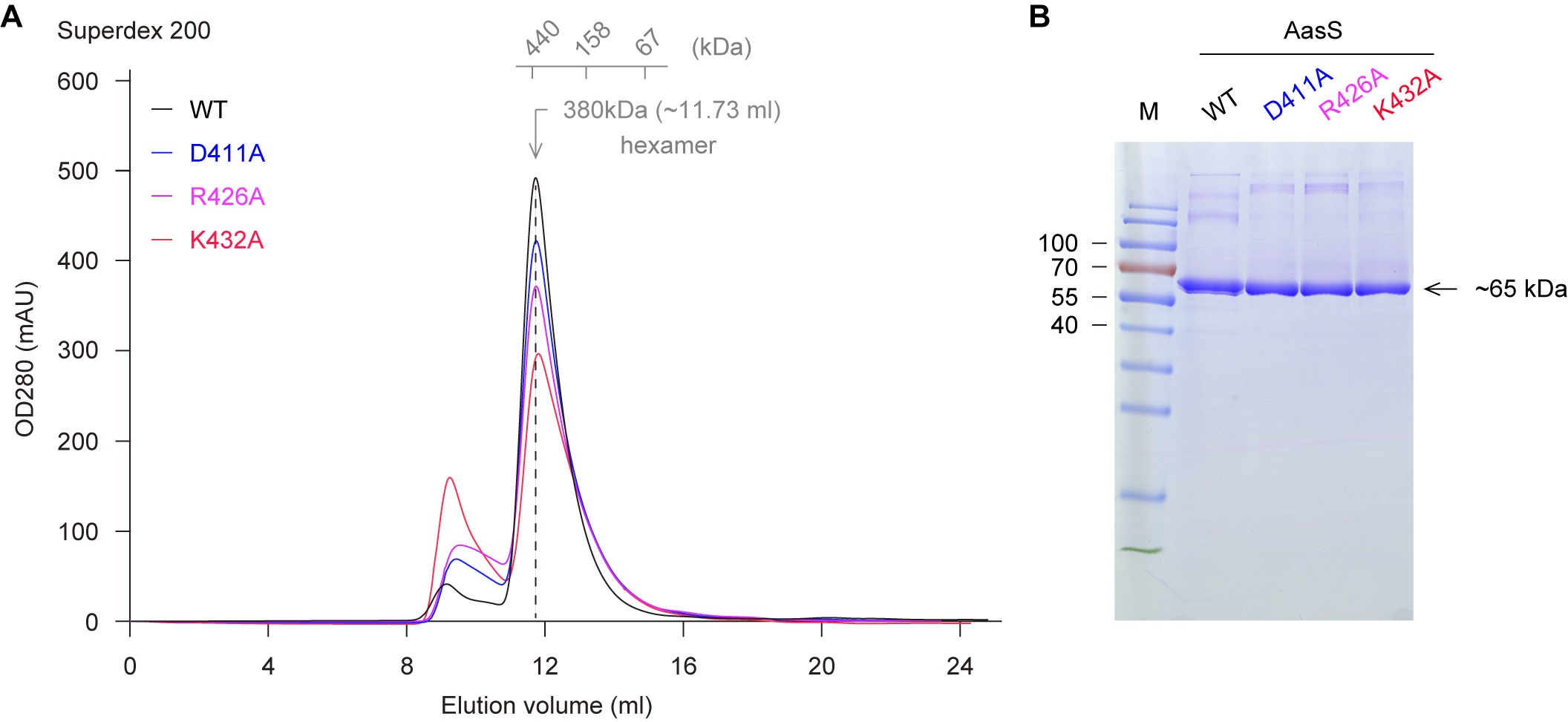

Supplement: S12 Fig — A. SEC analysis of AasS and three mutated versions. The SEC profile of AasS eluted at the position of ~11.73 ml suggested that all the three mutants retain the solution structure of hexamer. In addition to WT, the three AasS mutants included D411A, R426A, and K432A, respectively. B. SDS-PAGE (12%) profile of the purified AasS derivatives. The AasS protein of ~65 kDa was highlighted with an arrow. Abbreviations: SEC, Size exclusion chromatography; kDa, kilo-Dalton; M, protein marker. (TIF) [file ppat.1012376.s014.tif]

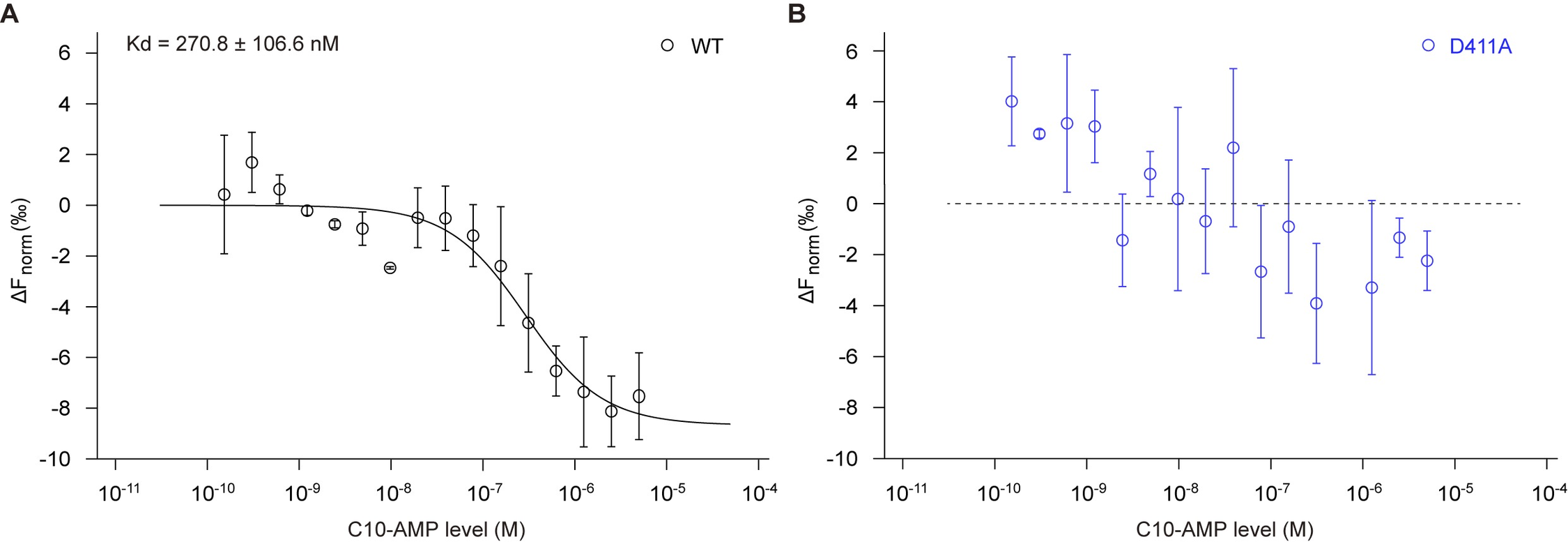

Supplement: S13 Fig — A. MST assays suggested efficient binding of C10 acyl adenylate by the wild-type AasS enzyme. B. The alanine substitution of D411A rendered AasS enzyme to lose its ability of binding C10 acyl adenylate in the MST experiments. The data was presented in mean ± SD (n, 3 independent trials). The data generally agreed with the conclusion by our ITC analyses. Abbreviations: MST, Microscale thermophoresis; ΔFnorm, the difference in normalized fluorescence against the concentration of its non-fluorescent ligand molecule. (TIF) [file ppat.1012376.s015.tif]
